# Complementary Health Approach Use Among Adults Reporting a Clinician Diagnosis of Chronic Fatigue Syndrome in a US Online Quota Sample

**DOI:** 10.3390/jcm15218425

**Published:** 2026-10-29

**Authors:** Kyle Agostini, Abgail N. Heims, John DiCristo, Andrew D. Bond, Robert N. Agnello, Thomas M. Motyka

**Affiliations:** 1 Jerry M. Wallace School of Osteopathic Medicine, Campbell University, Lillington, NC 27546, USA; 2 Department of Osteopathic Manipulative Medicine, Jerry M. Wallace School of Osteopathic Medicine, Campbell University, Lillington, NC 27546, USAmotyka@campbell.edu (T.M.M.)

**Keywords:** chronic fatigue syndrome, self-reported diagnosis, complementary health approaches, healthcare utilization, cross-sectional studies, exploratory analysis

## Abstract

**Background**/**Objectives**: We compared reported complementary health approach (CHA) use by self-reported clinician diagnosis of chronic fatigue syndrome (CFS), with favorability and expected helpfulness as additional exploratory outcomes. **Methods**: This secondary analysis included 3022 adults from a 2024 US nonprobability online quota sample. Diagnoses were not clinically verified, and formal ME/CFS criteria were not assessed. Logistic models adjusted for ten covariates, using Firth regression for sparse or unstable estimates. Revision-time Benjamini–Hochberg correction defined nine question-based families by outcome and approach grouping. **Results**: Among 167 respondents reporting CFS, adjusted odds of use were higher for physical (aOR 2.22, 95% CI 1.60–3.07), nutritional (2.25, 1.34–3.80), combined (1.89, 1.24–2.88), and systems approaches (1.99, 1.32–3.01); all q < 0.05. Nineteen individual CHAs and all seven conventional approaches met q < 0.05 for use. Any-CHA use was 98.2% versus 94.6% (aOR 2.46, 95% CI 0.97–8.91; q = 0.065). Adding the count of other self-reported clinician diagnoses attenuated category-use associations; their CIs included 1. Psychological-category favorability was lower using a mean >3.5 cutoff, but its FDR threshold status depended on midpoint handling. No expectancy association met q < 0.05. **Conclusions**: Respondents reporting CFS reported greater use of multiple approaches. Additional-diagnosis adjustment changed the estimates, but the count cannot distinguish morbidity from healthcare contact or diagnosis ascertainment. The findings do not establish CFS-specific treatment use, substitution, or effectiveness.

## 1. Introduction

Myalgic encephalomyelitis/chronic fatigue syndrome (ME/CFS) is a chronic, multisystem illness characterized by a substantial reduction in activity, post-exertional malaise, unrefreshing sleep, and either cognitive impairment or orthostatic intolerance [1,2]. In the 2021–2022 US National Health Interview Survey, 1.3% of adults reported having been told by a health professional that they had CFS or ME and still having the condition [1]. That estimate was based on self-report rather than independent clinical confirmation. Recorded incidence varied by age in a Norwegian registry study [3], and infectious onset is commonly reported [4,5]. Diagnosis can be challenging because there is no single confirmatory test and alternative diagnoses must be considered [5]. The personal and economic burden is substantial [5,6].

Management remains symptom-directed. The US Centers for Disease Control and Prevention states that there is no cure or approved treatment for ME/CFS, although individual symptoms can be managed [7]. Evidence concerning cognitive behavioral therapy (CBT) and graded exercise therapy comes from different sources that should be distinguished. Patient surveys describe perceived symptom changes and include reports of worsening after graded exercise [8]; these are not randomized treatment comparisons. A reanalysis of the PACE trial challenged its original recovery claims [9]. A broad intervention review identified promising results for CBT and graded exercise but emphasized methodological limitations [10]. A later review reported some short- to medium-term improvements after these interventions while noting heterogeneous case definitions and predominantly self-reported outcomes [11]. Current NICE guidance describes CBT as an optional aid to managing symptoms rather than a cure and advises against programs using fixed incremental increases in activity, including graded exercise therapy as defined in that guideline [12]. Clinical recommendations and reviews describe pharmacologic and nonpharmacologic management tailored to symptoms [5,13,14]. A Taiwanese insurance-claims study documented more frequent prescribing of several conventional treatments in its CFS cohort than in its comparison group [15]. A case series of low-dose naltrexone provided preliminary observations rather than controlled efficacy evidence [16]. Complementary approaches have been reported among people with chronic fatiguing illnesses [17], but a systematic review identified limited and methodologically weak evidence for complementary therapies [18]. Earlier prognosis research reported low full-recovery rates in cohorts followed without systematic intervention [19].

The principal objective of this exploratory analysis was to compare reported prior-year CHA use between respondents reporting a clinician diagnosis of chronic fatigue syndrome (CFS) and respondents not reporting that diagnosis. Conventional approaches were examined to provide context for the use pattern. Additional exploratory analyses assessed favorability among users and expected helpfulness among nonusers of each approach. A recent Austrian survey compared intervention use and perceived helpfulness in self-identified ME/CFS and depression [20]. Our comparison addresses a different descriptive question: how reported use differs by CFS diagnosis report within the same adult online quota sample, without requiring the comparison group to have a particular disease or to be healthy. The survey recorded CFS, not formal ME/CFS diagnostic criteria. This CFS-focused secondary analysis and its objectives were developed after collection of the parent survey data; no prospective hypotheses or prespecified primary endpoint are claimed.

## 2. Materials and Methods

### 2.1. Study Design and Participants

This exploratory secondary analysis used a cross-sectional online survey of US adults recruited through Qualtrics Panels (Qualtrics, Provo, UT, USA) in August 2024. Recruitment was nonprobability-based. Adults aged at least 18 years who lived in the United States and could complete an English-language survey were eligible. Quotas for age, sex assigned at birth, race/ethnicity, education, and region followed US Census benchmarks, as described in the parent survey [21]. Panelists could enroll while the relevant quota cell remained open. Quota alignment does not establish population representativeness or known inclusion probabilities. The anonymous, self-administered questionnaire assessed diagnosed conditions and health-approach use. Incomplete responses and failure of either of the two attention checks were exclusion criteria in the parent survey. Counts invited, screened, excluded because quotas were full, incomplete, or failing an attention check were not provided to the analytic team. Response and completion rates therefore could not be calculated. The delivered dataset contained 3022 complete responses; this secondary analysis excluded none. No survey or post-stratification weights were available or applied. Sex assigned at birth was modeled; gender identity was collected separately but was not modeled because the less frequent response categories were sparse. Details of panel-source recruitment and incentive payments were not documented in the materials available for this secondary analysis. The same parent survey supported a distinct chronic-pain analysis [21]. Reporting follows STROBE.

Group assignment used the item: “Do you currently have, or have you had in the past year, one of the following health conditions as diagnosed by a medical professional?” with the condition “Chronic fatigue syndrome” and yes/no responses. Throughout the results, “reported CFS” denotes a self-reported clinician diagnosis. Clinical records, symptom-based diagnostic criteria, and independent diagnostic verification were unavailable. Respondents not reporting this diagnosis constituted the comparison group; they could have other illnesses or undiagnosed CFS and were not healthy controls. The exact use question and recorded response anchors are provided in Supplement S1, Section S1.

### 2.2. Measures, Outcomes, and Intervention Classification

The survey assessed 35 approaches. Twenty-eight were classified as CHAs and grouped into nutritional, physical, psychological, combined, and systems categories using the parent survey’s adaptation of the National Center for Complementary and Integrative Health framework [21]. Seven were conventional comparators: prescription medication, over-the-counter medication, surgery, physical therapy, psychotherapy, prescription diet, and heat or cold therapy. Prescription diet was classified as conventional, whereas self-directed special diet was classified as complementary. These are analytic categories and do not establish efficacy or regulatory status. The complete item labels and category mapping are provided in Supplement S1, Table S1A; conventional approaches were excluded from CHA composites.

Individual use outcomes were yes/no reports of use in the past year. Category and overall CHA use equaled use of at least one constituent approach; they did not measure frequency or the number of approaches used. Individual favorability ratings were analyzed only among respondents who used that approach, and expected helpfulness only among respondents who had not used it. The six response options ranged from very unfavorable/unhelpful (1) to very favorable/helpful (6), with slightly unfavorable/unhelpful (3) and slightly favorable/helpful (4) straddling the midpoint. Individual ratings of 4–6 were coded positive and 1–3 negative. This binary contrast describes the side of the response scale, not the intensity of appraisal, and discards ordinal information.

For each respondent, category favorability was the arithmetic mean of available ratings for approaches they used within that category; category expectancy averaged available ratings for approaches they had not used. Overall composites applied the same rules across all 28 CHAs. Each mean was the sum of applicable ratings divided by their number, with equal weight per rated approach and equal weight per respondent in regression. A composite was missing only if no applicable rating was available. Composite means >3.5 were classified as positive and means ≤3.5 as not positive. This revision-time definition places only means above the mathematical midpoint on the favorable/helpful side; a mean exactly 3.5 may reflect mixed ratings and is not an individual neutral response. The earlier ≥3.5 definition is retained as a sensitivity analysis. The change was made after inspection of the earlier results and is not presented as prospectively specified. A respondent could contribute to both composites by using some constituent approaches and not others. The number and identity of rated approaches varied between respondents, so these composites were descriptive summaries rather than validated measures of general treatment attitudes. Their construction and rating counts are detailed in Supplement S1, Sections S1 and S5. The broad use question covered treatment of illness, health improvement, and health maintenance and included vitamins or minerals, prayer, and art, music, or dance. Any-CHA use should not be interpreted as complementary treatment specifically for CFS or compared directly with narrower survey definitions.

### 2.3. Statistical Analysis

Categorical sample characteristics were compared with Pearson chi-square tests without continuity correction, or Fisher’s exact test when any expected cell count was below five. Table 1 *p*-values are nominal and descriptive, without multiplicity adjustment. Logistic models estimated unadjusted odds ratios (ORs), adjusted odds ratios (aORs), and 95% confidence intervals (CIs) for reported CFS versus no reported CFS diagnosis. Adjusted models included insurance, sex assigned at birth, education, age (four levels), race/ethnicity (four levels), residence size (three levels), employment (three levels), poverty (three levels), region (four levels), and body mass index (four levels). Insurance indicated public or private coverage versus no coverage. These covariates span demographic, socioeconomic, healthcare-access, geographic, and anthropometric correlates of diagnosis reporting and health-approach use. They were included together to describe associations conditional on these measured dimensions, without outcome-driven variable selection. They do not constitute a validated causal adjustment set. Additional diagnoses and healthcare-utilization measures were collected in the parent survey but were not included in the original models; limited post hoc checks are described below. The models contain 21 covariate coefficients plus the CFS indicator and intercept. They estimate associations, not predictive performance or causal effects; ORs should not be interpreted as risk ratios, particularly for common outcomes.

Table 1 retains five descriptive race/ethnicity categories: Asian, non-Hispanic; White/Caucasian, non-Hispanic; Black/African American, non-Hispanic; Hispanic; and all additional categories. The last combines the original American Indian/Alaskan Native/additional-category response and declined-to-answer response. Regression used four levels, combining Asian and all-additional responses because of sparse cells; White/Caucasian, non-Hispanic was the reference. The other categorical coding and reference levels are supplied with the reproducibility files. Bold characteristic headings and light-gray shading identify demographic blocks; they do not denote statistical significance.

An initial maximum-likelihood logistic model was fitted for each adjusted contrast. The model was refitted using Firth penalized likelihood if an exposure-by-outcome cell contained fewer than five observations or the initial Wald OR confidence-limit ratio (upper divided by lower limit) exceeded 50, not 50%. Nonconvergence or a nonfinite or extreme exposure coefficient (absolute log OR > 20) also triggered refitting. These operational safeguards were intended to flag sparsity or instability; the cutoffs are pragmatic, not universal guarantees of adequate estimation. Twenty adjusted models used Firth regression. Their CIs came from profile penalized likelihood and their *p*-values from penalized likelihood-ratio tests; ordinary models used Wald CIs and tests [22]. Table S2 identifies every penalized model, its trigger, and the corresponding initial maximum-likelihood estimate where reportable. Penalization does not eliminate sparse-data uncertainty. No prospective prespecification is claimed for the threshold, refitting, or multiplicity choices in this post-collection analysis.

Benjamini–Hochberg false discovery rate (FDR) correction was applied to nine families defined by the questions being addressed [23]. Use, favorability, and expectancy were each separated into six category/overall CHA tests (five categories plus overall), 28 individual CHA-modality tests, and seven conventional-approach tests. These families distinguish any use or aggregate ratings by category, specific modalities, and conventional-care context; all 123 adjusted tests remain reported. This question-based grouping was adopted during revision after the original results were available, replacing the earlier pooled 102-test CHA correction and nominal conventional analyses. Historical pooled corrections are provided in Supplement S1 for transparency. Both nominal two-sided *p*-values and within-family BH-adjusted *p*-values—here, termed q-values—are reported. A q-value below 0.05 prioritizes an exploratory finding for follow-up; it is not confirmatory evidence or a paper-wide error-rate guarantee. The maximum variance inflation factor was 2.79, and the maximum for the CFS indicator was 1.31. Sample size was fixed by the parent survey, without additional recruitment or a prospective power calculation. Sparse outcome strata can limit precision despite the total sample size.

CFS status, use indicators, and the ten original covariates were complete for all 3022 respondents. Every use model therefore included the full sample. Eligibility for individual ratings was conditional on use or nonuse of that modality. For CHA models, including composites, favorability denominators ranged from 102 to 2864 and expectancy denominators from 734 to 3017. Corresponding conventional-comparator ranges were 336–2495 and 527–2686. Models used available complete cases without imputation. Group-specific event counts and denominators are displayed for every rating outcome in the corresponding outcome tables below; full six-level distributions appear in Table S3.

Post hoc supplementary analyses retained the original covariates and examined alternative rating estimands. Cumulative-logit models used the six ordered individual ratings, with separate favorability and expectancy families of 28 CHA or seven conventional tests. Linear models with HC3 standard errors compared undichotomized respondent-level composite means, with six tests per rating outcome; fractional means were not treated as six-level ordinal outcomes. A boundary sensitivity retained the historical composite definition of mean ≥ 3.5 using the same six-test families as the revised > 3.5 definition. Individual binary ratings were unchanged. These checks examine intensity or midpoint handling and do not replace the primary focus on reported use. Supplement S1 reports every sensitivity result, numerical checks, proportional-odds limitations, and historical correction schemes. Binary use outcomes do not have an ordinal sensitivity analysis.

We also fitted 26 supplementary use models: six overall/category CHA-use outcomes and seven conventional-use outcomes, each adding either a count of 18 other current/past-year self-reported clinician diagnoses or a categorical measure of past-year outpatient physician visits (0, 1–2, 3–5, 6–9, or ≥10). Within each specification, BH correction was applied separately to the six CHA and seven conventional tests. The count is an unvalidated summary of potentially overlapping diagnoses, not a measure of symptom severity; it may reflect morbidity, healthcare contact, diagnostic opportunity, and reporting. Physician visits may themselves follow illness and overlap conceptually with the use outcomes. These specifications describe additional conditional associations rather than corrected causal effects. Details are in Supplement S1, Section S6 and Table S9. Results were regenerated with Python 3.12.14, NumPy 2.3.5, pandas 2.2.3, and SciPy 1.17.0. The original 123-model outputs were reproduced before revision; all 12 affected composite-rating rows were then refitted, with counts and both crude and adjusted estimates regenerated. Code, data definitions, full-precision outputs, and numerical checks accompany the submission. No subgroup or interaction analyses were performed.

### 2.4. Use of Generative Artificial Intelligence

Claude (Anthropic, San Francisco, CA, USA) supported development of the earlier analysis pipeline. ChatGPT/Codex (OpenAI, San Francisco, CA, USA) assisted with revision, code development, numerical reconciliation, sensitivity analyses, and document, table, and figure preparation. AI assistance did not supply participant observations or clinical diagnoses. The authors are responsible for verifying the analyses, source interpretations, and final manuscript; tool-assisted numerical reproduction does not independently verify recruitment, diagnosis, or causal assumptions. Model/version identifiers for the AI sessions are not available in the retained project records.

## 3. Results

### 3.1. Sample Characteristics

Of 3022 respondents, 167 (5.5%) reported a clinician diagnosis of CFS and 2855 did not. Respondents assigned female at birth comprised 58.7% and 50.0% of the respective groups (nominal *p* = 0.029). The remaining Table 1 comparisons did not have nominal *p* < 0.05; this does not establish equivalence or absence of confounding. Quotas covered age, sex assigned at birth, race/ethnicity, education, and region and did not make the sample representative of US adults.

All 3022 delivered responses entered the analysis. Pre-delivery disposition counts were unavailable, precluding a complete recruitment flow or response-rate calculation. The original covariates and use indicators were complete, while rating denominators varied with modality-specific use and rating availability. In the parent data, the mean count of 18 other current/past-year self-reported clinician diagnoses was 5.67 in the reported-CFS group and 2.19 in the comparison group (Table S9).

### 3.2. Complementary Health Approach Use

**Figure 1:**
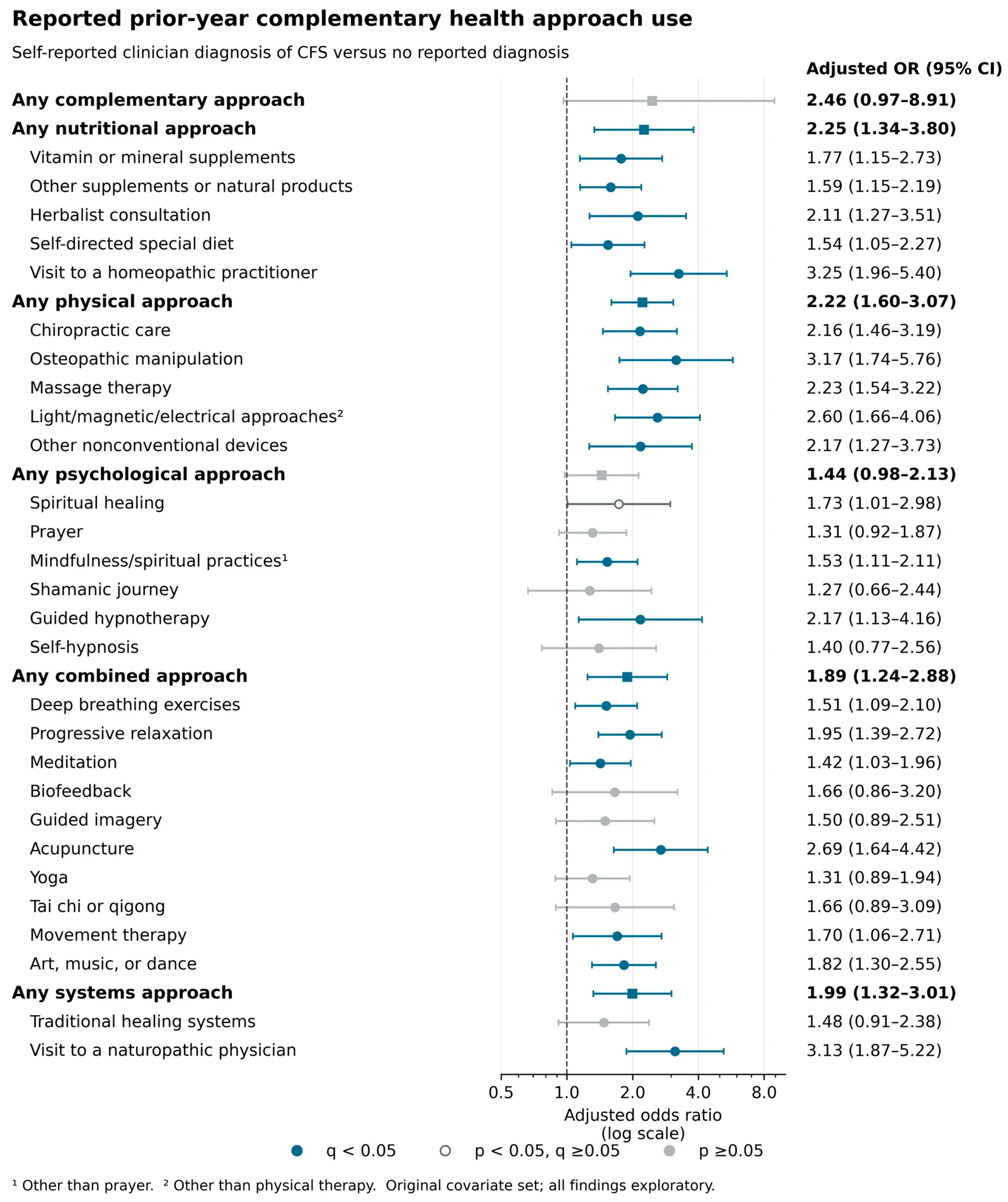
Adjusted odds ratios and 95% CIs for reported prior-year CHA use, comparing respondents reporting a clinician diagnosis of CFS with respondents not reporting that diagnosis. Formal ME/CFS criteria were not assessed. Models use the original covariate set and do not adjust for other reported conditions. Squares denote overall/category estimates, and circles denote individual-modality estimates. Filled blue markers denote q < 0.05 within the six-test category/overall-use or 28-test modality-use family; open gray markers denote nominal *p* < 0.05 with q ≥ 0.05; light-gray markers denote *p* ≥ 0.05. An FDR-prioritized exploratory finding is not confirmatory evidence. Use could concern any illness, health improvement, or maintenance. The axis is logarithmic; the vertical line denotes OR = 1. Figure S1 shows the category estimates with added other-diagnosis count or physician-visit category.

Under the original adjustment set, respondents reporting CFS had higher adjusted odds of use in four of five CHA categories (Table 2; Figure 1): physical (aOR 2.22, 95% CI 1.60–3.07), nutritional (2.25, 1.34–3.80), combined (1.89, 1.24–2.88), and systems approaches (1.99, 1.32–3.01), all q < 0.05. The psychological-category estimate was 1.44 (0.98–2.13; q = 0.065). These estimates concern any use within each category, not frequency, intensity, or CFS-specific treatment. The use findings were sensitive to the adjustment specification: after adding the count of other self-reported clinician diagnoses, the 95% confidence intervals included 1 for all six overall/category CHA-use outcomes and all seven conventional-use outcomes (Section 3.6; Table S9).

Reported use of at least one of the 28 broadly defined CHAs was 98.2% in the reported-CFS group and 94.6% in the comparison group (aOR 2.46, 95% CI 0.97–8.91; *p* = 0.0607; q = 0.065). The broad inclusion of health-maintenance practices and common approaches limits the interpretation of this high percentage. The estimate is imprecise and does not establish either a difference or equivalence between groups.

Nineteen of 28 individual CHA-use associations met q < 0.05 within the modality-use family. These included practitioner visits, physical approaches, supplements, dietary practices, and psychological or combined approaches (Table 2). For example, mindfulness or spiritual practices other than prayer had aOR 1.53 (95% CI 1.11–2.11; q = 0.019), and vitamin/mineral supplements had aOR 1.77 (1.15–2.73; q = 0.020).

### 3.3. Favorability of Approaches Used

Among users of each CHA, no modality-level favorability association met q < 0.05 in the 28-test family (Table 3). Mindfulness or spiritual practices other than prayer had aOR 0.37 (95% CI 0.19–0.70; *p* = 0.0024; q = 0.068). The naturopathic-physician-visit estimate was based on 20 favorable and one unfavorable response in the reported-CFS group (aOR 5.88, 95% CI 1.17–60.44; *p* = 0.0296; q = 0.277). Its very wide CI limits interpretation of the point estimate.

Using a mean >3.5 to define a favorable composite, the overall estimate was aOR 0.67 (95% CI 0.40–1.14; q = 0.216). The psychological composite had lower odds of a favorable mean (aOR 0.46, 95% CI 0.28–0.75; q = 0.013), but its FDR threshold status depended on the treatment of exact midpoint means (Section 3.6). These comparisons concern selected sets of users and their rated approaches, not general attitudes in the full diagnosis-report groups.

### 3.4. Expectations of Benefit

No overall, category-level, or individual CHA expected-helpfulness association met q < 0.05 (Table 4). Individual estimates compare nonusers of that approach. Composite estimates summarize each respondent’s available ratings of unused approaches, even if they used other approaches in the category. Failure to meet the FDR threshold does not establish equivalent expectations.

### 3.5. Conventional Comparator Analyses

Reported use percentages and adjusted odds were higher for all seven conventional approaches (Table 5). All seven use associations met q < 0.05 within the seven-test use family. Prescription medication (aOR 3.46, 95% CI 2.05–5.84), prescription diet (2.88, 2.00–4.15), psychotherapy (2.22, 1.48–3.33), physical therapy (2.22, 1.59–3.10), heat or cold therapy (2.18, 1.58–3.00), and surgery (2.04, 1.41–2.96) had q < 0.001. Over-the-counter medication use had an aOR of 1.67 (95% CI 1.00–2.78; *p* = 0.0496). This association met the q < 0.05 threshold under the revised seven-test use family (q = 0.0496), but not under the historical pooled 21-test conventional family (q = 0.116). Given the revision-time choice of testing families and this sensitivity to the multiplicity strategy, it should be interpreted as exploratory rather than as a robust independent finding. Among users, over-the-counter medication favorability was lower (0.51, 0.33–0.77; q = 0.010). No conventional expectancy association met q < 0.05; prescription-medication expectancy had aOR 0.29 (0.11–0.79; q = 0.107). Psychotherapy favorability was imprecisely estimated (3.21, 0.97–16.66; *p* = 0.057; q = 0.199). These outcomes describe use in the preceding year and selected rating subsets, not frequency of use.

### 3.6. Supplementary Analyses

Adding the count of 18 other self-reported clinician diagnoses attenuated the category-use estimates: physical aOR 1.27 (95% CI 0.89–1.82), nutritional 1.66 (0.97–2.85), combined 1.35 (0.87–2.09), and systems 1.24 (0.79–1.96). None of the six CHA composites or seven conventional-use outcomes had a CI excluding 1 in that specification. With physician-visit category added instead, physical, nutritional, combined, and systems CHA use and five conventional-use outcomes met q < 0.05 within the corresponding six- and seven-test families (Table S9; Figure S1). These conditional comparisons cannot identify why estimates changed.

All 70 six-level individual-rating models passed the stated numerical checks. Proportional odds could not be reliably assessed under the diagnostic screen for 47 models, including all favorability models. No CHA ordinal rating association met q < 0.05; mindfulness/spiritual-practice favorability had ordinal OR 0.52 (95% CI 0.33–0.80; q = 0.089). Among conventional ratings, only lower OTC-medication favorability met q < 0.05 (ordinal OR 0.59, 95% CI 0.44–0.81; q = 0.006). No undichotomized composite-mean contrast met q < 0.05. Psychological favorability had adjusted mean difference −0.289 (95% CI −0.512 to −0.066; q = 0.068). Under the historical composite cutoff ≥3.5, its binary estimate was aOR 0.53 (95% CI 0.30–0.94; q = 0.176), compared with 0.46 (0.28–0.75; q = 0.013) under >3.5, using the same six-test family. Thirty-three respondents, including six reporting CFS, had exact midpoint means. Supplement S1 provides complete results and historical pooled corrections.

## 4. Discussion

### 4.1. Principal Findings

Respondents reporting a clinician diagnosis of CFS had higher adjusted odds of reported use in four CHA categories, 19 individual CHA modalities, and all seven conventional approaches within the revised question-based testing families. Overall any-CHA use remained imprecisely estimated. This is a descriptive pattern in the sampled diagnosis-report groups, without evidence that the approaches were used specifically for CFS. Adding the count of other self-reported clinician diagnoses attenuated the category-use estimates; none of the 13 count-adjusted use CIs excluded 1. This sensitivity does not establish that other illness explains the associations: diagnosis count may reflect both morbidity and opportunities to obtain diagnoses through healthcare contact, as well as recall and reporting.

The 5.5% reporting CFS in this sample contrasts with the 1.3% NHIS estimate [1]. The discrepancy is substantial, but the definitions and sampling designs differ: NHIS asked about ever receiving a CFS/ME diagnosis and still having it, whereas this survey asked about a current or past-year CFS diagnosis. Both relied on self-report. Differential panel participation, diagnosis reporting, and case ascertainment could contribute; their separate contributions cannot be identified here. Demographic quota matching and covariate adjustment cannot repair unknown inclusion probabilities or unmeasured selection. Internet access, panel membership, and willingness to participate may also select respondents [24,25]. These findings should not be interpreted as US prevalence estimates or results from a clinically verified ME/CFS cohort.

Higher reported use of both complementary and conventional approaches provides context for the sampled groups’ healthcare contact. It does not establish visit frequency, continuity of follow-up, simultaneous or sequential use, or individual substitution. The diagnostic evaluation process could itself create opportunities to identify additional conditions, but these data do not establish the evaluation received by respondents or the timing of their diagnoses. The diagnosis count and conventional-use measures may therefore reflect some shared aspects of healthcare engagement. Symptom-management needs across several domains are another plausible explanation [5,26], but aggregate symptom severity was not measured. Physician-visit adjustment and diagnosis-count adjustment condition on related but distinct measures with uncertain temporal and causal roles; neither isolates a CFS-specific effect.

Favorability and expectancy were additional outcomes with limited evidence of broad differences. No individual CHA rating association met the revised FDR threshold in either binary or ordinal analyses. Psychological-category favorability met that threshold only under the revised > 3.5 binary cutoff, not under the historical ≥ 3.5 rule or the undichotomized mean-score analysis. The negative direction was consistent, but the threshold-dependent finding should not be treated as robust evidence of a general attitude difference. Selection into use creates different user subsets in the two diagnosis-report groups, and demographic adjustment does not remove that problem. Composite means already divide by the number of applicable ratings, but their composition differs: overall favorability summarized a median of six approaches in respondents reporting CFS and five in the comparison group. These summaries do not measure effectiveness or a common latent treatment attitude.

The use findings are compatible with earlier reports of substantial complementary-approach use in chronic fatiguing illnesses [17]. Other research has described varied motivations for seeking complementary practitioners [27], but those motivations were not measured in this survey and should not be attributed to its respondents. The broad list, including vitamin/mineral supplements, prayer, and art, music, or dance for treatment or health maintenance, also helps explain the high any-CHA percentage. The measured construct is broader than complementary medicine used specifically to treat CFS.

Dorczok et al. compared intervention use and perceived helpfulness in self-identified ME/CFS and depression [20]. Our analysis instead compares respondents reporting and not reporting a CFS diagnosis within a US online quota sample, uses an explicit demographic and socioeconomic adjustment set, and examines expected helpfulness among modality-specific nonusers. These differences define the contribution and prevent direct equivalence between the studies’ populations or estimands. Neither cross-sectional survey determines therapeutic efficacy or individual treatment substitution.

Use associations for practitioner visits, physical practices, or supplements identify areas for further descriptive research. They do not justify recommending an approach: efficacy, safety, and cost-effectiveness require evidence beyond utilization, and the cited systematic review of complementary therapies identified important evidentiary limitations [18].

### 4.2. Clinical Implications

One potential clinical consideration is to ask patients about the full range of medicines, supplements, diets, practices, and devices they use, together with the intended purpose, perceived benefit, adverse effects, cost, and timing. The observed use pattern may inform such conversations, but this study did not test whether reconciliation improves outcomes or whether any reported approach is effective. Decisions about care should be grounded in intervention-specific evidence and individual circumstances.

For clinically assessed ME/CFS, discussions of physical activity should account for post-exertional malaise and individual tolerance, consistent with clinical guidance [5,12]. The survey’s physical and combined categories are heterogeneous and should not be interpreted as exercise prescriptions or as evidence supporting graded exercise therapy.

### 4.3. Strengths and Limitations

Strengths include explicit exposure and outcome definitions, a common sampling setting for the two comparison groups, disclosure of the quota dimensions, reporting of denominators and full-precision estimates, sparse-data safeguards, and multiplicity adjustment. Numerical reproduction and supplementary analyses make the effects of analytic choices visible. They do not establish clinical validity or eliminate observational biases.

The leading limitations are the unverified self-reported CFS diagnosis, absence of formal ME/CFS criteria, nonprobability recruitment, and unavailable recruitment-disposition counts. The comparison group is not a healthy population. Only 167 respondents reported CFS, with still smaller user and nonuser subsets for many ratings; Firth penalization does not guarantee precise estimates. CFS severity and duration were not available, and the original adjustment set omitted other reported conditions and healthcare utilization. The diagnosis-count and visit analyses demonstrate sensitivity to added adjustment but use incomplete measures with uncertain causal roles; diagnosis count is not a validated measure of illness or symptom severity. Low VIFs do not resolve this conceptual uncertainty. Multiple tests remain exploratory, and FDR control applies within each named family, not across the paper. The family definitions and composite cutoff were revised after earlier results were known, creating additional scope for analytic flexibility; historical comparisons are retained. Dichotomization discards rating intensity, and composite threshold status can depend on exact midpoint handling. Averaging assumes equal numerical spacing and combines varying numbers and types of approaches; conditioning ratings on use can create selection bias. The survey did not establish approach-specific indication, motivation, frequency, dose, cost, adverse effects, or simultaneous use. Finally, the cross-sectional design cannot establish temporal ordering or causation, and past-year recall and self-report may introduce error.

### 4.4. Research Priorities

Replication should use probability-based sampling or clearly defined clinical cohorts with verified ME/CFS criteria and measures of severity, duration, comorbid conditions, healthcare access, and treatment timing. Longitudinal studies could distinguish adoption, persistence, and substitution and relate them to patient-reported outcomes and adverse effects. Research should also account for inequities in access to diagnosis [28,29]. Future rating analyses could model repeated ratings while accounting for modality identity and respondent-level dependence; prospective justification of the target estimand and outcome hierarchy would strengthen interpretation.

## 5. Conclusions

In this US nonprobability online quota sample, respondents reporting an unverified clinician diagnosis of CFS reported greater use of multiple complementary and conventional approaches under the original covariate set. These descriptive associations do not establish CFS-specific treatment use. Adjustment for other self-reported clinician diagnoses attenuated estimates, but that count cannot separate morbidity from healthcare contact and diagnosis ascertainment. Secondary rating findings were limited and, for psychological-category favorability, cutoff-dependent. The findings may inform conversations about reported health-approach use but do not establish treatment preferences, substitution, effectiveness, or applicability to clinically verified ME/CFS.

## Figures and Tables

**Table 1:** Sample characteristics by self-reported clinician diagnosis of CFS. CFS reported and CFS not reported refer to the diagnosis item, not clinical confirmation or health status. All *p*-values are nominal and unadjusted for multiplicity. Race/ethnicity has five descriptive categories; regression uses four, as described in Section 2.3. No values are missing for the characteristics shown.

Characteristic	CFS Not Reported n (%)	CFS Reported n (%)	Total n (%)	Nominal *p*
All respondents	2855 (94.5)	167 (5.5)	3022	
**Age**				**0.424**
18–34	842 (29.5)	48 (28.7)	890 (29.5)	
35–49	704 (24.7)	44 (26.3)	748 (24.8)	
50–64	713 (25.0)	48 (28.7)	761 (25.2)	
65 and older	596 (20.9)	27 (16.2)	623 (20.6)	
**Sex assigned at birth**				**0.029**
Male	1428 (50.0)	69 (41.3)	1497 (49.5)	
Female	1427 (50.0)	98 (58.7)	1525 (50.5)	
**Race/Ethnicity**				**0.253**
Asian, non-Hispanic	145 (5.1)	4 (2.4)	149 (4.9)	
White/Caucasian, non-Hispanic	1749 (61.3)	102 (61.1)	1851 (61.3)	
Black/African American, non-Hispanic	311 (10.9)	14 (8.4)	325 (10.8)	
Hispanic	512 (17.9)	36 (21.6)	548 (18.1)	
All additional categories	138 (4.8)	11 (6.6)	149 (4.9)	
**Education**				**0.239**
Less than college	1067 (37.4)	70 (41.9)	1137 (37.6)	
Some college or more	1788 (62.6)	97 (58.1)	1885 (62.4)	
**Employment**				**0.776**
Not working	1494 (52.3)	85 (50.9)	1579 (52.3)	
Part-time	389 (13.6)	26 (15.6)	415 (13.7)	
Full-time	972 (34.0)	56 (33.5)	1028 (34.0)	
**Income**				**0.354**
In poverty	813 (28.5)	56 (33.5)	869 (28.8)	
Near poverty	325 (11.4)	19 (11.4)	344 (11.4)	
Above poverty	1717 (60.1)	92 (55.1)	1809 (59.9)	
**Insurance**				**0.084**
Uninsured	309 (10.8)	11 (6.6)	320 (10.6)	
Insured	2546 (89.2)	156 (93.4)	2702 (89.4)	
**Region**				**0.174**
East	496 (17.4)	19 (11.4)	515 (17.0)	
Midwest	605 (21.2)	35 (21.0)	640 (21.2)	
South	1085 (38.0)	74 (44.3)	1159 (38.4)	
West	669 (23.4)	39 (23.4)	708 (23.4)	
**Residence size**				**0.259**
City	1480 (51.8)	85 (50.9)	1565 (51.8)	
Town	844 (29.6)	43 (25.7)	887 (29.4)	
Village or countryside	531 (18.6)	39 (23.4)	570 (18.9)	
**Body mass index**				**0.376**
Underweight	126 (4.4)	5 (3.0)	131 (4.3)	
Normal	995 (34.9)	58 (34.7)	1053 (34.8)	
Overweight	798 (28.0)	40 (24.0)	838 (27.7)	
Obese	936 (32.8)	64 (38.3)	1000 (33.1)	

**Table 2:** Reported prior-year CHA use by self-reported clinician diagnosis of CFS.

Approach	CFS Not Reported n/N (%)	CFS Reported n/N (%)	Crude OR (95% CI)	Adjusted OR (95% CI)	*p*	q
**Any complementary approach †**	**2700/2855** **(94.6)**	**164/167** **(98.2)**	**3.14 (0.99–9.94)**	**2.46 (0.97–8.91)**	**0.061**	**0.065**
**Any nutritional approach**	**2288/2855** **(80.1)**	**150/167** **(89.8)**	**2.19 (1.31–3.64)**	**2.25 (1.34–3.80)**	**0.002**	**0.004**
Vitamin or mineral supplements	2148/2855 (75.2)	140/167 (83.8)	1.71 (1.12–2.60)	1.77 (1.15–2.73)	0.010	0.020
Other dietary supplements or natural products	1247/2855 (43.7)	91/167 (54.5)	1.54 (1.13–2.11)	1.59 (1.15–2.19)	0.005	0.012
Herbalist consultation	181/2855 (6.3)	20/167 (12.0)	2.01 (1.23–3.28)	2.11 (1.27–3.51)	0.004	0.011
Self-directed special diet	447/2855 (15.7)	37/167 (22.2)	1.53 (1.05–2.24)	1.54 (1.05–2.27)	0.028	0.043
Visit to a homeopathic practitioner	133/2855 (4.7)	21/167 (12.6)	2.94 (1.80–4.80)	3.25 (1.96–5.40)	<0.001	<0.001
**Any physical approach**	**763/2855** **(26.7)**	**74/167** **(44.3)**	**2.18 (1.59–2.99)**	**2.22 (1.60–3.07)**	**<0.001**	**<0.001**
Chiropractic care	331/2855 (11.6)	37/167 (22.2)	2.17 (1.48–3.18)	2.16 (1.46–3.19)	<0.001	<0.001
Osteopathic manipulation	87/2855 (3.0)	15/167 (9.0)	3.14 (1.77–5.56)	3.17 (1.74–5.76)	<0.001	<0.001
Massage therapy	445/2855 (15.6)	47/167 (28.1)	2.12 (1.49–3.02)	2.23 (1.54–3.22)	<0.001	<0.001
Light, magnetic, or electrical approaches other than physical therapy	199/2855 (7.0)	27/167 (16.2)	2.57 (1.66–3.98)	2.60 (1.66–4.06)	<0.001	<0.001
Other nonconventional devices	151/2855 (5.3)	17/167 (10.2)	2.03 (1.20–3.44)	2.17 (1.27–3.73)	0.005	0.012
**Any psychological approach**	**2033/2855** **(71.2)**	**132/167** **(79.0)**	**1.52 (1.04–2.23)**	**1.44 (0.98–2.13)**	**0.065**	**0.065**
Spiritual healing	179/2855 (6.3)	17/167 (10.2)	1.69 (1.00–2.86)	1.73 (1.01–2.98)	0.0472	0.066
Prayer	1829/2855 (64.1)	119/167 (71.3)	1.39 (0.99–1.96)	1.31 (0.92–1.87)	0.135	0.151
Mindfulness or spiritual practices other than prayer	1029/2855 (36.0)	77/167 (46.1)	1.52 (1.11–2.08)	1.53 (1.11–2.11)	0.009	0.019
Shamanic journey	148/2855 (5.2)	11/167 (6.6)	1.29 (0.68–2.43)	1.27 (0.66–2.44)	0.470	0.470
Guided hypnotherapy	101/2855 (3.5)	12/167 (7.2)	2.11 (1.14–3.92)	2.17 (1.13–4.16)	0.020	0.034
Self-hypnosis	171/2855 (6.0)	13/167 (7.8)	1.32 (0.74–2.38)	1.40 (0.77–2.56)	0.271	0.281
**Any combined approach**	**2030/2855** **(71.1)**	**138/167** **(82.6)**	**1.93 (1.29–2.91)**	**1.89 (1.24–2.88)**	**0.003**	**0.004**
Deep breathing exercises	1391/2855 (48.7)	100/167 (59.9)	1.57 (1.14–2.16)	1.51 (1.09–2.10)	0.013	0.024
Progressive relaxation	686/2855 (24.0)	62/167 (37.1)	1.87 (1.35–2.59)	1.95 (1.39–2.72)	<0.001	<0.001
Meditation	1174/2855 (41.1)	83/167 (49.7)	1.41 (1.04–1.93)	1.42 (1.03–1.96)	0.031	0.0462
Biofeedback	119/2855 (4.2)	11/167 (6.6)	1.62 (0.86–3.07)	1.66 (0.86–3.20)	0.133	0.151
Guided imagery	218/2855 (7.6)	18/167 (10.8)	1.46 (0.88–2.43)	1.50 (0.89–2.51)	0.129	0.151
Acupuncture	167/2855 (5.8)	22/167 (13.2)	2.44 (1.52–3.93)	2.69 (1.64–4.42)	<0.001	<0.001
Yoga	562/2855 (19.7)	40/167 (24.0)	1.29 (0.89–1.86)	1.31 (0.89–1.94)	0.176	0.190
Tai chi or qigong	140/2855 (4.9)	12/167 (7.2)	1.50 (0.81–2.77)	1.66 (0.89–3.09)	0.112	0.142
Movement therapy	247/2855 (8.7)	23/167 (13.8)	1.69 (1.07–2.67)	1.70 (1.06–2.71)	0.026	0.043
Art, music, or dance	1418/2855 (49.7)	108/167 (64.7)	1.86 (1.34–2.57)	1.82 (1.30–2.55)	<0.001	0.002
**Any systems approach**	**339/2855** **(11.9)**	**33/167** **(19.8)**	**1.83 (1.23–2.72)**	**1.99 (1.32–3.01)**	**0.001**	**0.003**
Traditional healing systems	284/2855 (9.9)	22/167 (13.2)	1.37 (0.86–2.19)	1.48 (0.91–2.38)	0.111	0.142
Visit to a naturopathic physician	135/2855 (4.7)	21/167 (12.6)	2.90 (1.78–4.73)	3.13 (1.87–5.22)	<0.001	<0.001

Note: Values are users/eligible respondents (%); all use denominators are 2855 and 167. Bold type and light-gray shading identify overall and category summary rows; they do not denote statistical significance. These rows summarize any use within a category or overall. Adjusted models include the ten original covariates in Section 2.3. *p* is the adjusted-model Wald *p*-value, or penalized likelihood-ratio *p*-value for Firth models (†); q is BH-adjusted separately across six category/overall-use tests and 28 individual modality-use tests. Crude ORs use the unadjusted two-by-two table. CIs are Wald intervals except profile penalized-likelihood intervals for adjusted Firth estimates. The approaches were not necessarily used for CFS. Abbreviations: OR, odds ratio; CI, confidence interval; CFS, reported clinician diagnosis of chronic fatigue syndrome.

**Table 3:** Favorable ratings among users of each CHA and dichotomized respondent-level mean ratings for category and overall composites.

Approach	CFS Not Reported n/N (%)	CFS Reported n/N (%)	Crude OR (95% CI)	Adjusted OR (95% CI)	*p*	q
**Overall CHA mean rating**	**2505/2700** **(92.8)**	**147/164** **(89.6)**	**0.67 (0.40–1.14)**	**0.67 (0.40–1.14)**	**0.144**	**0.216**
**Nutritional mean rating**	**2019/2288** **(88.2)**	**133/150** **(88.7)**	**1.04 (0.62–1.75)**	**1.16 (0.68–1.98)**	**0.579**	**0.579**
Vitamin or mineral supplements	1978/2148 (92.1)	129/140 (92.1)	1.01 (0.53–1.90)	1.11 (0.58–2.13)	0.747	0.951
Other dietary supplements or natural products	1071/1247 (85.9)	81/91 (89.0)	1.33 (0.68–2.62)	1.60 (0.79–3.23)	0.193	0.624
Herbalist consultation †	142/181 (78.5)	18/20 (90.0)	2.47 (0.55–11.11)	2.34 (0.67–12.69)	0.200	0.624
Self-directed special diet	361/447 (80.8)	31/37 (83.8)	1.23 (0.50–3.04)	1.39 (0.53–3.64)	0.498	0.742
Visit to a homeopathic practitioner †	102/133 (76.7)	20/21 (95.2)	6.08 (0.78–47.13)	4.19 (0.87–41.84)	0.077	0.359
**Physical mean rating**	**665/763** **(87.2)**	**67/74** **(90.5)**	**1.41 (0.63–3.16)**	**1.57 (0.68–3.61)**	**0.286**	**0.344**
Chiropractic care †	299/331 (90.3)	34/37 (91.9)	1.21 (0.35–4.17)	0.93 (0.32–3.63)	0.907	0.988
Osteopathic manipulation †	63/87 (72.4)	13/15 (86.7)	2.48 (0.52–11.80)	3.22 (0.73–23.74)	0.131	0.522
Massage therapy †	408/445 (91.7)	45/47 (95.7)	2.04 (0.48–8.75)	1.60 (0.49–8.15)	0.468	0.742
Light, magnetic, or electrical approaches other than physical therapy	163/199 (81.9)	21/27 (77.8)	0.77 (0.29–2.05)	0.64 (0.19–2.16)	0.468	0.742
Other nonconventional devices	118/151 (78.1)	12/17 (70.6)	0.67 (0.22–2.04)	0.36 (0.07–1.96)	0.236	0.661
**Psychological mean rating**	**1862/2033** **(91.6)**	**110/132** **(83.3)**	**0.46 (0.28–0.74)**	**0.46 (0.28–0.75)**	**0.002**	**0.013**
Spiritual healing †	145/179 (81.0)	14/17 (82.4)	1.09 (0.30–4.02)	1.63 (0.45–7.66)	0.477	0.742
Prayer	1718/1829 (93.9)	107/119 (89.9)	0.58 (0.31–1.08)	0.55 (0.29–1.05)	0.070	0.359
Mindfulness or spiritual practices other than prayer	947/1029 (92.0)	63/77 (81.8)	0.39 (0.21–0.73)	0.37 (0.19–0.70)	0.002	0.068
Shamanic journey †	104/148 (70.3)	11/11 (100.0)	NE	8.24 (0.98–1072.78)	0.0526	0.359
Guided hypnotherapy †	77/101 (76.2)	8/12 (66.7)	0.62 (0.17–2.25)	0.56 (0.15–2.33)	0.416	0.742
Self-hypnosis †	132/171 (77.2)	11/13 (84.6)	1.63 (0.35–7.64)	2.11 (0.51–12.33)	0.316	0.738
**Combined mean rating**	**1893/2030** **(93.3)**	**124/138** **(89.9)**	**0.64 (0.36–1.14)**	**0.64 (0.36–1.16)**	**0.143**	**0.216**
Deep breathing exercises †	1298/1391 (93.3)	96/100 (96.0)	1.72 (0.62–4.78)	1.48 (0.62–4.57)	0.412	0.742
Progressive relaxation	629/686 (91.7)	57/62 (91.9)	1.03 (0.40–2.68)	1.00 (0.37–2.69)	0.998	0.998
Meditation	1086/1174 (92.5)	77/83 (92.8)	1.04 (0.44–2.45)	0.91 (0.38–2.19)	0.831	0.988
Biofeedback †	94/119 (79.0)	9/11 (81.8)	1.20 (0.24–5.89)	0.92 (0.19–5.97)	0.921	0.988
Guided imagery †	190/218 (87.2)	16/18 (88.9)	1.18 (0.26–5.40)	1.43 (0.35–8.35)	0.638	0.850
Acupuncture	129/167 (77.2)	15/22 (68.2)	0.63 (0.24–1.66)	0.72 (0.24–2.16)	0.555	0.777
Yoga	521/562 (92.7)	33/40 (82.5)	0.37 (0.15–0.89)	0.35 (0.14–0.90)	0.030	0.277
Tai chi or qigong †	122/140 (87.1)	9/12 (75.0)	0.44 (0.11–1.79)	0.60 (0.14–2.91)	0.503	0.742
Movement therapy †	212/247 (85.8)	19/23 (82.6)	0.78 (0.25–2.44)	0.91 (0.30–3.32)	0.880	0.988
Art, music, or dance †	1351/1418 (95.3)	106/108 (98.1)	2.63 (0.64–10.88)	1.84 (0.62–9.01)	0.308	0.738
**Systems mean rating †**	**271/339** **(79.9)**	**31/33** **(93.9)**	**3.89 (0.91–16.65)**	**2.93 (0.91–14.83)**	**0.074**	**0.216**
Traditional healing systems †	240/284 (84.5)	19/22 (86.4)	1.16 (0.33–4.09)	1.04 (0.33–4.28)	0.952	0.988
Visit to a naturopathic physician †	96/135 (71.1)	20/21 (95.2)	8.13 (1.05–62.65)	5.88 (1.17–60.44)	0.030	0.277

Note: Values are positive ratings/eligible respondents (%), with explicit group-specific denominators. Individual positivity means a rating of 4–6; composite positivity means an available-rating mean > 3.5. Category and overall rows summarize different sets and numbers of used approaches across respondents; they do not mean that every constituent approach was rated favorably. BH correction applies separately to six category/overall-favorability tests and 28 individual modality-favorability tests. Other conventions, covariates, and Firth markers are as in Table 2. NE: crude OR and Wald CI not reported because of a zero cell. Bold type and light-gray shading identify overall and category summary rows; they do not denote statistical significance. † Firth penalized likelihood with profile penalized-likelihood 95% CIs and penalized likelihood-ratio *p*-values.

**Table 4:** Positive expected helpfulness among nonusers of each CHA and dichotomized respondent-level mean expectations for category and overall composites.

Approach	CFS Not Reported n/N (%)	CFS Reported n/N (%)	Crude OR (95% CI)	Adjusted OR (95% CI)	*p*	q
**Overall CHA mean rating**	**1055/2850** **(37.0)**	**64/167** **(38.3)**	**1.06 (0.77–1.46)**	**1.06 (0.76–1.47)**	**0.725**	**0.847**
**Nutritional mean rating**	**1205/2821** **(42.7)**	**58/160** **(36.2)**	**0.76 (0.55–1.06)**	**0.76 (0.55–1.07)**	**0.116**	**0.694**
Vitamin or mineral supplements	451/707 (63.8)	14/27 (51.9)	0.61 (0.28–1.32)	0.61 (0.27–1.37)	0.232	0.663
Other dietary supplements or natural products	987/1608 (61.4)	41/76 (53.9)	0.74 (0.46–1.17)	0.77 (0.48–1.23)	0.276	0.663
Herbalist consultation	1175/2674 (43.9)	57/147 (38.8)	0.81 (0.57–1.14)	0.83 (0.58–1.17)	0.279	0.663
Self-directed special diet	1258/2408 (52.2)	58/130 (44.6)	0.74 (0.52–1.05)	0.75 (0.52–1.08)	0.118	0.663
Visit to a homeopathic practitioner	1077/2722 (39.6)	56/146 (38.4)	0.95 (0.67–1.34)	0.93 (0.66–1.32)	0.686	0.873
**Physical mean rating**	**1296/2843** **(45.6)**	**75/166** **(45.2)**	**0.98 (0.72–1.35)**	**0.97 (0.70–1.34)**	**0.847**	**0.847**
Chiropractic care	1495/2524 (59.2)	72/130 (55.4)	0.85 (0.60–1.22)	0.84 (0.59–1.21)	0.355	0.663
Osteopathic manipulation	1243/2768 (44.9)	62/152 (40.8)	0.85 (0.61–1.18)	0.84 (0.60–1.18)	0.322	0.663
Massage therapy	1612/2410 (66.9)	78/120 (65.0)	0.92 (0.63–1.35)	0.94 (0.63–1.39)	0.742	0.873
Light, magnetic, or electrical approaches other than physical therapy	1072/2656 (40.4)	57/140 (40.7)	1.01 (0.72–1.43)	1.01 (0.71–1.44)	0.941	0.980
Other nonconventional devices	1014/2704 (37.5)	63/150 (42.0)	1.21 (0.86–1.68)	1.20 (0.86–1.69)	0.284	0.663
**Psychological mean rating**	**752/2830** **(26.6)**	**47/165** **(28.5)**	**1.10 (0.78–1.56)**	**1.10 (0.77–1.57)**	**0.605**	**0.847**
Spiritual healing	957/2676 (35.8)	59/150 (39.3)	1.16 (0.83–1.63)	1.18 (0.84–1.66)	0.350	0.663
Prayer	359/1026 (35.0)	12/48 (25.0)	0.62 (0.32–1.21)	0.64 (0.32–1.27)	0.201	0.663
Mindfulness or spiritual practices other than prayer	772/1826 (42.3)	35/90 (38.9)	0.87 (0.56–1.34)	0.88 (0.56–1.36)	0.553	0.873
Shamanic journey	718/2707 (26.5)	41/156 (26.3)	0.99 (0.68–1.42)	1.00 (0.68–1.45)	0.980	0.980
Guided hypnotherapy	938/2754 (34.1)	59/155 (38.1)	1.19 (0.85–1.66)	1.18 (0.84–1.66)	0.327	0.663
Self-hypnosis	709/2684 (26.4)	42/154 (27.3)	1.04 (0.73–1.50)	1.03 (0.71–1.49)	0.875	0.980
**Combined mean rating**	**1130/2847** **(39.7)**	**60/165** **(36.4)**	**0.87 (0.63–1.20)**	**0.88 (0.63–1.23)**	**0.464**	**0.847**
Deep breathing exercises	815/1464 (55.7)	31/67 (46.3)	0.69 (0.42–1.12)	0.73 (0.44–1.21)	0.219	0.663
Progressive relaxation	1162/2169 (53.6)	54/105 (51.4)	0.92 (0.62–1.36)	0.92 (0.62–1.38)	0.693	0.873
Meditation	882/1681 (52.5)	38/84 (45.2)	0.75 (0.48–1.16)	0.79 (0.50–1.23)	0.295	0.663
Biofeedback	867/2736 (31.7)	55/156 (35.3)	1.17 (0.84–1.65)	1.19 (0.85–1.68)	0.309	0.663
Guided imagery	802/2637 (30.4)	44/149 (29.5)	0.96 (0.67–1.38)	0.94 (0.65–1.36)	0.747	0.873
Acupuncture	1184/2688 (44.0)	61/145 (42.1)	0.92 (0.66–1.29)	0.94 (0.67–1.33)	0.748	0.873
Yoga	1190/2293 (51.9)	63/127 (49.6)	0.91 (0.64–1.30)	0.94 (0.65–1.35)	0.731	0.873
Tai chi or qigong	1157/2715 (42.6)	61/155 (39.4)	0.87 (0.63–1.22)	0.89 (0.64–1.25)	0.501	0.873
Movement therapy	1249/2608 (47.9)	65/144 (45.1)	0.90 (0.64–1.25)	0.94 (0.67–1.33)	0.743	0.873
Art, music, or dance	709/1437 (49.3)	25/59 (42.4)	0.75 (0.45–1.28)	0.75 (0.44–1.28)	0.291	0.663
**Systems mean rating**	**1018/2775** ** (36.7)**	**55/157** **(35.0)**	**0.93 (0.66–1.30)**	**0.93 (0.66–1.31)**	**0.671**	**0.847**
Traditional healing systems	1142/2571 (44.4)	54/145 (37.2)	0.74 (0.53–1.05)	0.75 (0.52–1.06)	0.103	0.663
Visit to a naturopathic physician	1095/2720 (40.3)	59/146 (40.4)	1.01 (0.72–1.41)	1.01 (0.71–1.42)	0.959	0.980

Note: Values are positive expectations/eligible respondents (%). Individual positivity means a rating of 4–6 among nonusers of that modality; composite positivity means a mean > 3.5 across available ratings of unused constituent approaches. Thus a composite denominator is not restricted to people who used no approaches in that category. BH correction applies separately to six category/overall-expectancy tests and 28 individual modality-expectancy tests. Other conventions are as in Table 2. Bold type and light-gray shading identify overall and category summary rows; they do not denote statistical significance.

**Table 5:** Conventional approaches by self-reported clinician diagnosis of CFS, with separate seven-test FDR families for use, favorability, and expectancy.

Approach	CFS Not Reported n/N (%)	CFS Reported n/N (%)	Crude OR (95% CI)	Adjusted OR (95% CI)	*p*	q
**Reported use in the past year**						
Prescription medication	2054/2855 (71.9)	150/167 (89.8)	3.44 (2.07–5.72)	3.46 (2.05–5.84)	<0.001	<0.001
Over-the-counter medication	2346/2855 (82.2)	149/167 (89.2)	1.80 (1.09–2.96)	1.67 (1.00–2.78)	0.0496	0.0496
Surgery	404/2855 (14.2)	42/167 (25.1)	2.04 (1.42–2.94)	2.04 (1.41–2.96)	<0.001	<0.001
Physical therapy	585/2855 (20.5)	61/167 (36.5)	2.23 (1.61–3.10)	2.22 (1.59–3.10)	<0.001	<0.001
Psychotherapy	301/2855 (10.5)	35/167 (21.0)	2.25 (1.52–3.33)	2.22 (1.48–3.33)	<0.001	<0.001
Prescription diet	349/2855 (12.2)	48/167 (28.7)	2.90 (2.03–4.12)	2.88 (2.00–4.15)	<0.001	<0.001
Heat or cold therapy	983/2855 (34.4)	90/167 (53.9)	2.23 (1.63–3.05)	2.18 (1.58–3.00)	<0.001	<0.001
**Favorable ratings among users**						
Prescription medication	1765/2054 (85.9)	131/150 (87.3)	1.13 (0.69–1.86)	1.18 (0.71–1.95)	0.525	0.616
Over-the-counter medication	2054/2346 (87.6)	116/149 (77.9)	0.50 (0.33–0.75)	0.51 (0.33–0.77)	0.001	0.010
Surgery	325/404 (80.4)	34/42 (81.0)	1.03 (0.46–2.32)	1.25 (0.52–3.01)	0.616	0.616
Physical therapy	493/585 (84.3)	50/61 (82.0)	0.85 (0.43–1.69)	0.83 (0.40–1.70)	0.606	0.616
Psychotherapy †	248/301 (82.4)	33/35 (94.3)	3.53 (0.82–15.15)	3.21 (0.97–16.66)	0.057	0.199
Prescription diet	280/349 (80.2)	41/48 (85.4)	1.44 (0.62–3.36)	2.21 (0.89–5.45)	0.087	0.202
Heat or cold therapy	911/983 (92.7)	82/90 (91.1)	0.81 (0.38–1.74)	0.78 (0.35–1.73)	0.540	0.616
**Positive expected helpfulness among nonusers**						
Prescription medication	601/801 (75.0)	9/17 (52.9)	0.37 (0.14–0.98)	0.29 (0.11–0.79)	0.015	0.107
Over-the-counter medication	319/509 (62.7)	10/18 (55.6)	0.74 (0.29–1.92)	0.64 (0.24–1.73)	0.380	0.686
Surgery	1472/2451 (60.1)	75/125 (60.0)	1.00 (0.69–1.44)	1.06 (0.73–1.54)	0.761	0.815
Physical therapy	1535/2270 (67.6)	66/106 (62.3)	0.79 (0.53–1.18)	0.84 (0.55–1.26)	0.392	0.686
Psychotherapy	1031/2554 (40.4)	56/132 (42.4)	1.09 (0.76–1.55)	1.12 (0.78–1.60)	0.556	0.779
Prescription diet	1649/2506 (65.8)	72/119 (60.5)	0.80 (0.55–1.16)	0.82 (0.56–1.21)	0.316	0.686
Heat or cold therapy	1117/1872 (59.7)	44/77 (57.1)	0.90 (0.57–1.43)	0.95 (0.59–1.51)	0.815	0.815

Note: Values are positive outcomes/eligible respondents (%). Use is prior-year use for any reported health purpose; favorability concerns users and expected helpfulness concerns nonusers of that modality. Ratings 4–6 are positive. The q column applies BH correction separately within the seven conventional models for each outcome type. † Firth penalized likelihood with profile CIs and likelihood-ratio *p*-value; other adjusted models use maximum likelihood and Wald inference. No association is confirmatory. Bold type and light-gray shading identify outcome-type headings (use, favorability, and expectancy); they do not denote statistical significance.

## Data Availability

The de-identified respondent-level analytic dataset, variable dictionary, and original analysis code are publicly available at https://github.com/kagostini1127-collab/CFS_CHA (accessed on 21 October 2026). Supplement S2 provides the aligned derived diagnosis-count and physician-visit input, code, and full-precision outputs for the revised analyses. The full parent survey dataset is not included in these public files; requests for additional parent-survey data may be directed to Dr. Thomas M. Motyka (motyka@campbell.edu), subject to the applicable data-governance terms.

## Supplementary Materials

The following supporting information can be downloaded at: https://www.mdpi.com/article/10.3390/jcm15218425/s1, Supplement S1 contains measurement documentation, Sections S1–S7, the supplementary tables, and Figure S1. Table S1. Complete revised binary-model inventory for reported use, favorability among users, and expected helpfulness among nonusers. Table S1A. Recorded modality labels and analytic classification. Table S1B. Historical composite-cutoff estimates with historical pooled and revised question-based multiplicity corrections. Table S1C. Comparison of inference under historical correction, changed testing families alone, and the final revised analysis. Table S2. Firth penalized-likelihood estimates and initial maximum-likelihood alternatives for all 20 penalized adjusted models. Table S3. Six-level individual-rating distributions by reported CFS diagnosis and outcome. Table S4. Adjusted cumulative-logit models for all 70 six-level individual-rating outcomes. Table S5. Adjusted differences in undichotomized respondent-level category and overall composite means. Table S6. Composite-rating sensitivity to exact-midpoint handling under matched six-test families. Table S7. Exposure-focused proportional-odds diagnostics and reasons for nonassessment. Table S8. Number and composition of ratings contributing to respondent-level composites. Table S9. Descriptive summaries and use associations after separate additional adjustment for other reported diagnoses or physician-visit category. Figure S1. Overall and category CHA-use estimates under the original covariates, with added other-condition count, and with added physician-visit category. CFS refers to a self-reported clinician diagnosis. Horizontal bars are 95% CIs; OR >1 indicates higher odds of reported use. The post hoc specifications do not identify causal effects or establish control of symptom severity or diagnosis ascertainment. Supplement S2 is the reproducibility archive containing the analytic inputs, analysis code, variable definitions, numerical checks, full-precision table files, and figure files for the revised analyses. The completed STROBE checklist is supplied as nonpublished reporting material. Supplement S2 is provided in related link https://github.com/kagostini1127-collab/CFS_CHA (accessed on 21 October 2026).

## References

[1] Vahratian A., Lin J.M.S., Bertolli J., Unger E.R. (2023). Myalgic Encephalomyelitis/Chronic Fatigue Syndrome in Adults: United States, 2021–2022. NCHS Data Brief..

[2] Institute of Medicine (2015). Beyond Myalgic Encephalomyelitis/Chronic Fatigue Syndrome: Redefining an Illness.

[3] Bakken I.J., Tveito K., Gunnes N., Ghaderi S., Stoltenberg C., Trogstad L., Åberg S.E.H., Magnus P. (2014). Two age peaks in the incidence of chronic fatigue syndrome/myalgic encephalomyelitis: A population-based registry study from Norway 2008–2012. BMC Med..

[4] Naess H., Sundal E., Myhr K.M., Nyland H.I. (2010). Postinfectious and chronic fatigue syndromes: Clinical experience from a tertiary-referral centre in Norway. In Vivo.

[5] Bateman L., Bested A.C., Bonilla H.F., Chheda B.V., Chu L., Curtin J.M., Dempsey T.T., Dimmock M.E., Dowell T.G., Felsenstein D. (2021). Myalgic Encephalomyelitis/Chronic Fatigue Syndrome: Essentials of Diagnosis and Management. Mayo Clin. Proc..

[6] Lin J.M.S., Resch S.C., Brimmer D.J., Johnson A., Kennedy S., Burstein N., Simon C.J. (2011). The economic impact of chronic fatigue syndrome in Georgia: Direct and indirect costs. Cost Eff. Resour. Alloc..

[7] Centers for Disease Control and Prevention Manage ME/CFS. Updated 10 May 2024. https://www.cdc.gov/me-cfs/management/index.html.

[8] Geraghty K., Hann M., Kurtev S. (2019). Myalgic encephalomyelitis/chronic fatigue syndrome patients’ reports of symptom changes following cognitive behavioural therapy, graded exercise therapy and pacing treatments: Analysis of a primary survey compared with secondary surveys. J. Health Psychol..

[9] Wilshire C.E., Kindlon T., Courtney R., Matthees A., Tuller D., Geraghty K., Levin B. (2018). Rethinking the treatment of chronic fatigue syndrome—A reanalysis and evaluation of findings from a recent major trial of graded exercise and CBT. BMC Psychol..

[10] Whiting P., Bagnall A.M., Sowden A.J., Cornell J.E., Mulrow C.D., Ramírez G. (2001). Interventions for the Treatment and Management of Chronic Fatigue Syndrome: A Systematic Review. JAMA.

[11] Ingman T., Smakowski A., Goldsmith K., Chalder T. (2022). A systematic literature review of randomized controlled trials evaluating prognosis following treatment for adults with chronic fatigue syndrome. Psychol. Med..

[12] National Institute for Health and Care Excellence (2021). Myalgic Encephalomyelitis (or Encephalopathy)/Chronic Fatigue Syndrome: Diagnosis and Management. NICE Guideline NG206. https://www.nice.org.uk/guidance/ng206/chapter/recommendations.

[13] Castro-Marrero J., Sáez-Francàs N., Santillo D., Alegre J. (2017). Treatment and management of chronic fatigue syndrome/myalgic encephalomyelitis: All roads lead to Rome. Br. J. Pharmacol..

[14] ME/CFS Clinician Coalition (2021). ME/CFS Treatment Recommendations, Version 1. https://mecfscliniciancoalition.org/wp-content/uploads/2021/05/MECFS-Clinician-Coalition-Treatment-Recs-V1.pdf.

[15] Leong K.H., Yip H.T., Kuo C.F., Tsai S.Y. (2022). Treatments of chronic fatigue syndrome and its debilitating comorbidities: A 12-year population-based study. J. Transl. Med..

[16] Bolton M.J., Chapman B.P., Van Marwijk H. (2020). Low-dose naltrexone as a treatment for chronic fatigue syndrome. BMJ Case Rep..

[17] Jones J.F., Maloney E.M., Boneva R.S., Jones A.B., Reeves W.C. (2007). Complementary and alternative medical therapy utilization by people with chronic fatiguing illnesses in the United States. BMC Complement Altern. Med..

[18] Alraek T., Lee M.S., Choi T.Y., Cao H., Liu J. (2011). Complementary and alternative medicine for patients with chronic fatigue syndrome: A systematic review. BMC Complement Altern. Med..

[19] Cairns R., Hotopf M. (2005). A systematic review describing the prognosis of chronic fatigue syndrome. Occup. Med..

[20] Dorczok M.C., Mossaheb N., Mittmann G., Thomas M.F., Bartova L., Schrank B., Steiner-Hofbauer V. (2026). Use and perceived helpfulness of different intervention strategies in myalgic encephalomyelitis/chronic fatigue syndrome and depression. J. Clin. Med..

[21] Agostini K., Acheson A.D., Agnello R., Ortiz R., Motyka T.M. (2025). The association of chronic pain and complementary health approach usage: A comprehensive analysis adjusting for demographics. J. Pain Res..

[22] Heinze G., Schemper M. (2002). A solution to the problem of separation in logistic regression. Stat. Med..

[23] Benjamini Y., Hochberg Y. (1995). Controlling the false discovery rate: A practical and powerful approach to multiple testing. J. R. Stat. Soc. Ser. B Methodol..

[24] Owen J.E., Bantum E.O., Criswell K., Bazzo J., Gorlick A., Stanton A.L. (2014). Representativeness of two sampling procedures for an internet intervention targeting cancer-related distress: A comparison of convenience and registry samples. J. Behav. Med..

[25] Wright K.B. (2005). Researching Internet-Based Populations: Advantages and Disadvantages of Online Survey Research, Online Questionnaire Authoring Software Packages, and Web Survey Services. J. Comput.-Mediat. Commun..

[26] Castro-Marrero J., Faro M., Aliste L., Sáez-Francàs N., Calvo N., Martínez-Martínez A., de Sevilla T.F., Alegre J. (2017). Comorbidity in Chronic Fatigue Syndrome/Myalgic Encephalomyelitis: A Nationwide Population-Based Cohort Study. Psychosomatics.

[27] Sirois F.M. (2008). Motivations for consulting complementary and alternative medicine practitioners: A comparison of consumers from 1997–8 and 2005. BMC Complement Altern. Med..

[28] Samms G.L., Ponting C.P. (2025). Unequal access to diagnosis of myalgic encephalomyelitis in England. BMC Public Health.

[29] Jones C.L., Younger J. (2025). Possible Racial Disparities in the Diagnosis of Myalgic Encephalomyelitis/Chronic Fatigue Syndrome (ME/CFS). Int. J. Environ. Res. Public Health.

