# Supplementary material for: Complementary Health Approach Use Among Adults Reporting a Clinician Diagnosis of Chronic Fatigue Syndrome in a US Online Quota Sample
Source: J Clin Med. 2026 Oct 29;15(21):8425. doi: 10.3390/jcm15218425 (PMC13763643; doi:10.3390/jcm15218425)

Supplement S1

Measurement documentation and exploratory supplementary analyses

Complementary Health Approach Use among Adults Reporting a Clinician Diagnosis of Chronic Fatigue Syndrome in a US Online Quota Sample

This supplement documents the survey-defined diagnosis, outcome construction, numerical methods, and post hoc checks accompanying the revised manuscript. The revised 123 binary models are the main analyses. The additional results qualify the interpretation and are not a prospectively specified confirmatory analysis.

S1 Measurement and outcome construction

Diagnosis item

Do you currently have, or have you had in the past year, one of the following health conditions as diagnosed by a medical professional? - Chronic fatigue syndrome

Responses: No (0); Yes (1). A yes response records a reported diagnosis made by a medical professional, currently or in the past year. It does not verify symptoms, diagnostic criteria, or medical records. Respondents answering no are not a healthy control group.

Use item

Please indicate whether you have used each of the following conventional or non-conventional approaches to treat an illness, improve health, or maintain health in the last year?

Each modality has its own yes/no field: "No, have not used in the past year" (0) and "Yes, have used in the past year" (1). Respondents can report using multiple modalities. The documented item list and recorded rating anchors follow. The dictionary does not preserve the complete introductory stems of the favorability and expected-helpfulness blocks, the visual presentation order or randomization, or an other/specify option. Piloting, validation, panel-source recruitment, and incentive details were not documented in the materials available for this secondary analysis. The recorded coding order should not be treated as proof of screen presentation order.

Recorded six point response anchors

Code	Favorability	Expected helpfulness
1	Very unfavorable	Very unhelpful
2	Somewhat unfavorable	Somewhat unhelpful
3	Slightly unfavorable	Slightly unhelpful
4	Slightly favorable	Slightly helpful
5	Somewhat favorable	Somewhat helpful
6	Very favorable	Very helpful

Individual ratings 4–6 are positive and 1–3 negative. Applicable ratings are summed and divided by their number within each respondent; the mean already accounts for the number of ratings. Dividing again would change the score incorrectly. Favorability includes used approaches only; expectancy includes unused approaches only. The revised binary composite is positive only when the mean is >3.5. Exactly 3.5 is classified as not positive, which can reflect a balance of favorable and unfavorable ratings rather than an unfavorable appraisal. No applicable rating means missing. Respondents receive equal weight, although their means vary in item composition. These are descriptive summaries, not validated attitude measures. Use composites mean any use, not an average or count.

The modality classification in Table S1A follows the parent survey and is retained for continuity. It is not a judgment of efficacy, safety, professional regulation, or whether every use of an approach is complementary in a particular clinical setting.

S2 Statistical specifications and numerical reconciliation

The principal focus is reported use, with favorability and expectancy as additional exploratory outcomes. During revision, BH correction was reorganized into nine question-based families: six category/overall CHA tests, 28 individual CHA tests, and seven conventional tests, separately for use, favorability, and expectancy. Category families include all five categories plus overall. All 123 adjusted contrasts remain in Table S1. These families distinguish aggregate/category questions, specific modalities, and

conventional-care context. They control multiplicity within families, not jointly across the paper. The choices were made after earlier results were known and are not prospectively prespecified. Table S1B supplies the original-cutoff results with historical pooled corrections (102 CHA and 21 conventional tests) and the revised question-based corrections; Table S1C separates changes due to family definition from those due to midpoint handling. Conventional q21 values were themselves first added during revision; the submitted manuscript reported nominal conventional p-values.

All adjusted models include the original 21 covariate coefficients plus the reported-CFS indicator and intercept. The original 123-model output was reproduced before revision. Earlier discrepancies in sparse-model intervals and p-values were corrected independently of the later cutoff and family changes. The 12 composite-rating rows were then fully refitted under >3.5, including counts, percentages, and crude and adjusted inference; all other estimates are unchanged. Naturopathic-physician favorability remains imprecise: aOR 5.88 (95% CI 1.17–60.44; revised q = 0.277). Psychotherapy favorability is aOR 3.21 (0.97–16.66; p = 0.057; revised q = 0.199). Table S1C records the complete inference comparison rather than highlighting only findings that cross 0.05.

Firth refitting was triggered by any diagnosis-by-outcome cell <5, an initial maximum-likelihood Wald upper/lower OR confidence-limit ratio >50, nonconvergence, or an exposure log OR that was nonfinite or had absolute value >20. Twenty adjusted models used Firth estimation, with profile penalized-likelihood CIs and penalized likelihood-ratio tests. Table S2 includes the initial maximum-likelihood alternatives. Of 19 reportable exposure coefficients, 17 had the same direction. Chiropractic and biofeedback favorability crossed OR = 1 between methods; the shamanic-journey ML estimate was not reportable. The alternatives are not a gold standard in sparse data. Low VIFs or converged likelihoods do not ensure precise or unbiased estimates.

Multiplicity in supplementary analyses

The 70 ordinal models are corrected within four families: CHA favorability (28), CHA expectancy (28), conventional favorability (7), and conventional expectancy (7). The 12 undichotomized composite models use separate six-test favorability and expectancy families. Historical ≥3.5 composite estimates are corrected within the same six-test families as revised >3.5 estimates, isolating midpoint handling. The diagnosis-count and physician-visit specifications each use a six-test CHA-use family and a seven-test conventional-use family. Use is binary and has no ordinal sensitivity. Earlier rating-only, matched-inventory, and pooled q26 calculations are retained in the historical reproducibility files but do not determine current inferential claims. Sensitivities address alternative estimands or conditioning sets and are not independent confirmations. No minimum q-value is selected across correction schemes. All tests remain two-sided.

S3 Six level individual ratings

For each approach, an unpenalized cumulative-logit model retained the six ordered response levels and the original covariates. An OR >1 denotes higher odds of a higher rating under the proportional-odds assumption, a different estimand from crossing the original positive-rating boundary. Table S3 supplies all response-level counts, and Table S4 supplies all 70 model results. We did not apply the binary Firth implementation to ordinal outcomes.

The implementation used SciPy optimization with an analytic gradient and ordered cutpoints. Numerical admissibility required agreement of two starting values in log likelihood (<0.00001), maximum absolute score <0.0001, a positive numerical Hessian with minimum eigenvalue >0.0000001 and condition number <10 billion, finite standard errors, and absolute slopes <20. All 70 models passed these checks. The optimizer flag alone was not the criterion because precision-loss flags can coexist with sufficiently small scores. The gradient was checked numerically, the two-category likelihood was checked against binary logistic regression, and three selected models were reproduced using a separate direct-probability parameterization. These checks were performed within the supplied Python workflow; cross-software validation is not claimed. Wald CIs remain vulnerable to sparse-data bias.

An exposure-focused partial-proportional-odds likelihood comparison was attempted only when every diagnosis-by-rating cell contained at least five observations. This conservative screen permitted 23 expectancy diagnostics and excluded 47 models, including every favorability model. Three diagnostic tests had nominal p <0.05: expectations for osteopathic manipulation, massage, and surgery. These diagnostic p-values were not multiplicity-adjusted; they are flags rather than definitive assumption tests. Failure to assess the assumption is not evidence that it holds, and the diagnostic does not assess proportional odds for every covariate. Full results are in Table S7.

Mindfulness/spiritual-practice favorability had ordinal OR 0.52 (95% CI 0.33–0.80; p = 0.00319; q = 0.089), with the same direction as the binary OR of 0.37 (q = 0.068). No individual CHA rating outcome met q <0.05 in either analysis. Naturopathic-physician favorability had ordinal OR 1.45 (0.60–3.52), illustrating its imprecision. Among conventional ratings, lower OTC-medication favorability persisted (ordinal OR 0.59, 0.44–0.81; q = 0.006). Prescription-medication expectancy had ordinal OR 0.33 (0.15–0.76; q = 0.063), and no expectancy item met q <0.05. These are within-question comparisons using the stated family sizes, without mixing use tests into rating families.

S4 Composite means and exact midpoint sensitivity

Category and overall means can take fractional values, so they were not forced into six ordinal categories. Linear models estimated reported-CFS minus comparison-group differences in mean scores, using the original covariates, HC3 standard errors, and t-based intervals with n minus the 23 fitted coefficients as degrees of freedom. Means assume equal numerical spacing between response levels and summarize different sets of approaches. These analyses do not measure therapeutic benefit or a common latent attitude.

No composite mean contrast met q <0.05 within its six-test family. Psychological favorability had adjusted mean difference −0.289 (95% CI −0.512 to −0.066; q = 0.068). Overall favorability was −0.134 points (−0.282 to 0.014; q = 0.225), and overall expectancy was −0.030 (−0.220 to 0.160; q = 0.755). These estimates do not establish equivalence.

The revised main analysis uses mean >3.5 because only means above the midpoint are on the favorable/helpful side. This definition was adopted after the earlier results had been inspected; the historical ≥3.5 definition is retained as a sensitivity, not concealed. Individual integer ratings are unaffected. With the same six-test favorability family, psychological favorability changes from aOR 0.53 (95% CI 0.30–0.94; q = 0.176) under ≥3.5 to 0.46 (0.28–0.75; q = 0.013) under >3.5. Thirty-three respondents, including six reporting CFS, had means exactly 3.5. The direction is consistent, but threshold status depends on midpoint handling; the mean-score sensitivity also remains above q =0.05. No other composite rating meets q <0.05. Table S6 reports all 12 comparisons and midpoint counts.

S5 Number and composition of ratings

Table S8 describes how many ratings contribute to each respondent-level composite, by reported diagnosis. Overall favorability had a median of six ratings (IQR 4–10) among respondents reporting CFS versus five (3–8) in the comparison group. Overall expectancy had medians of 22 (18.5–24) and 24 (21–26), respectively. Within-group Spearman correlations between rating count and overall favorability mean were 0.135 and −0.019; corresponding expectancy correlations were −0.436 and −0.297. These are descriptive, unadjusted associations without hypothesis-test claims. They may reflect which approaches are used or left unused as well as the number rated. The arithmetic mean already divides by the rating count. We did not divide again, weight respondents by their rating count, or reinterpret the composites as standardized cross-modality attitudes.

S6 Other reported conditions and physician visits

The parent data were matched to the 3,022-row analytic extract using all 116 shared numerical fields. There were 3,021 distinct full patterns, with one pair identical on every shared field; those two parent rows are exchangeable for these models. All shared values matched. Only the derived count and visit category, aligned to the existing anonymous public row order, are included in the supplementary analytic input. The full parent data are not redistributed.

The unweighted condition count includes Acute COVID-19, Alzheimer's disease or other dementia, Asthma, Cancer, Chronic kidney disease, Chronic lung disease, Chronic pain, Diabetes (type I or type II), Epilepsy or seizures, Fibromyalgia, Heart disease, High cholesterol, Hypertension or high blood pressure, Long COVID-19, Low back pain, Migraine headaches, Rheumatoid arthritis, Stroke. The focal CFS item and the physical-disability indicator were excluded. Lifetime psychiatric diagnosis items were excluded because their reference period differs. The score has 18 possible items, enters the model as one linear count term, and is not a validated comorbidity or severity index. It includes overlapping conditions (for example, chronic pain and low back pain) and acute COVID-19, so it should not be called a chronic-disease count. All contributing fields were complete.

Past-year outpatient physician visits were coded 0, 1–2, 3–5, 6–9, and 10 or more, using four indicator terms with zero visits as the reference. The top category was not treated as an exact count of 10. Each specification separately augments the original covariates for six CHA-use composites and seven conventional-use outcomes. The covariate specifications were written before fitting those additional models, but the question-based correction families were adopted later during revision. BH correction now applies separately to six CHA and seven conventional tests within each specification; historical pooled q26 values remain in the CSV for provenance. No simultaneous count-plus-visits model or individual-diagnosis selection was performed.

The mean count of other self-reported clinician diagnoses was 5.67 versus 2.19, with medians 5 (IQR 3–7) versus 2 (1–3), for respondents reporting and not reporting CFS. After adding the count, none of the 13 use CIs excluded 1. Adding visit category retained four CHA-category and five conventional-use associations at q <0.05. The count may reflect morbidity, healthcare contact, diagnostic opportunity, and reporting; it does not measure aggregate symptom severity. Diagnostic evaluation could itself increase opportunities to identify other conditions, but the survey does not establish respondents' diagnostic workups or temporal ordering. Count and conventional-use measures may capture related aspects of healthcare engagement. Attenuation cannot establish that illness burden explains the association or quantify confounding. Conditioning can also change ORs for reasons other than confounding. In the count-adjusted use models, VIF was 1.15 for CFS and 1.25 for the diagnosis count (maximum 2.07); their correlation was 0.339. Low VIFs do not resolve the variables' conceptual or causal roles.

S7 Reproducibility and file inventory

The original de-identified analytic dataset, variable dictionary, and original code are publicly available at https://github.com/kagostini1127-collab/CFS_CHA. Supplement S2 contains the code and analytic inputs for the revised analyses, including the aligned derived diagnosis-count and physician-visit variables, full-precision CSV files for Tables S1–S9, numerical checks, and figure source files. The full parent survey dataset is not redistributed. The rendered tables below report all focal model contrasts and distributions; the CSV files additionally retain full-precision estimates, counts, and diagnostic fields. Rounded display values use two decimals for ORs and CIs, one for percentages, and three for p/q values, with four decimals near 0.05. Full precision determines threshold status. Mean-score differences use three decimals. Sources for survey wording are the original manuscript and parent SPSS variable/value labels, not reconstructed questionnaire screens. The numerical checks do not independently validate survey procedures or model assumptions.

Table S1. Complete revised binary-model inventory for reported use, favorability among users, and expected helpfulness among nonusers.

Adjusted models include the original ten covariates. CFS n/total gives the model-specific number reporting CFS and total N. Individual rating positivity is 4–6; composite positivity is mean >3.5. q uses the nine question-based families. † Firth penalized likelihood with profile intervals and penalized likelihood-ratio p-values; otherwise maximum likelihood and Wald inference. Crude estimates use two-by-two tables; NE denotes a nonreportable crude estimate.

Approach/composite	Outcome	CFS n/total	Crude OR (95% CI)	Adjusted OR (95% CI)	p	q
Overall CHA †	Use	167/3,022	3.14 (0.99–9.94)	2.46 (0.97–8.91)	0.061	0.065
Overall CHA	Favorability	164/2,864	0.67 (0.40–1.14)	0.67 (0.40–1.14)	0.144	0.216
Overall CHA	Expectancy	167/3,017	1.06 (0.77–1.46)	1.06 (0.76–1.47)	0.725	0.847
Nutritional	Use	167/3,022	2.19 (1.31–3.64)	2.25 (1.34–3.80)	0.002	0.004
Nutritional	Favorability	150/2,438	1.04 (0.62–1.75)	1.16 (0.68–1.98)	0.579	0.579
Nutritional	Expectancy	160/2,981	0.76 (0.55–1.06)	0.76 (0.55–1.07)	0.116	0.694
Physical	Use	167/3,022	2.18 (1.59–2.99)	2.22 (1.60–3.07)	<0.001	<0.001
Physical	Favorability	74/837	1.41 (0.63–3.16)	1.57 (0.68–3.61)	0.286	0.344
Physical	Expectancy	166/3,009	0.98 (0.72–1.35)	0.97 (0.70–1.34)	0.847	0.847
Psychological	Use	167/3,022	1.52 (1.04–2.23)	1.44 (0.98–2.13)	0.065	0.065
Psychological	Favorability	132/2,165	0.46 (0.28–0.74)	0.46 (0.28–0.75)	0.002	0.013
Psychological	Expectancy	165/2,995	1.10 (0.78–1.56)	1.10 (0.77–1.57)	0.605	0.847
Combined	Use	167/3,022	1.93 (1.29–2.91)	1.89 (1.24–2.88)	0.003	0.004
Combined	Favorability	138/2,168	0.64 (0.36–1.14)	0.64 (0.36–1.16)	0.143	0.216
Combined	Expectancy	165/3,012	0.87 (0.63–1.20)	0.88 (0.63–1.23)	0.464	0.847
Systems	Use	167/3,022	1.83 (1.23–2.72)	1.99 (1.32–3.01)	0.001	0.003
Systems †	Favorability	33/372	3.89 (0.91–16.65)	2.93 (0.91–14.83)	0.074	0.216
Systems	Expectancy	157/2,932	0.93 (0.66–1.30)	0.93 (0.66–1.31)	0.671	0.847
Vitamin or mineral supplements	Use	167/3,022	1.71 (1.12–2.60)	1.77 (1.15–2.73)	0.010	0.020
Vitamin or mineral supplements	Favorability	140/2,288	1.01 (0.53–1.90)	1.11 (0.58–2.13)	0.747	0.951
Vitamin or mineral supplements	Expectancy	27/734	0.61 (0.28–1.32)	0.61 (0.27–1.37)	0.232	0.663
Other dietary supplements or natural products	Use	167/3,022	1.54 (1.13–2.11)	1.59 (1.15–2.19)	0.005	0.012
Other dietary supplements or natural products	Favorability	91/1,338	1.33 (0.68–2.62)	1.60 (0.79–3.23)	0.193	0.624
Other dietary supplements or natural products	Expectancy	76/1,684	0.74 (0.46–1.17)	0.77 (0.48–1.23)	0.276	0.663
Herbalist consultation	Use	167/3,022	2.01 (1.23–3.28)	2.11 (1.27–3.51)	0.004	0.011
Herbalist consultation †	Favorability	20/201	2.47 (0.55–11.11)	2.34 (0.67–12.69)	0.200	0.624
Herbalist consultation	Expectancy	147/2,821	0.81 (0.57–1.14)	0.83 (0.58–1.17)	0.279	0.663
Self-directed special diet	Use	167/3,022	1.53 (1.05–2.24)	1.54 (1.05–2.27)	0.028	0.043
Self-directed special diet	Favorability	37/484	1.23 (0.50–3.04)	1.39 (0.53–3.64)	0.498	0.742
Self-directed special diet	Expectancy	130/2,538	0.74 (0.52–1.05)	0.75 (0.52–1.08)	0.118	0.663
Visit to a homeopathic practitioner	Use	167/3,022	2.94 (1.80–4.80)	3.25 (1.96–5.40)	<0.001	<0.001
Visit to a homeopathic practitioner †	Favorability	21/154	6.08 (0.78–47.13)	4.19 (0.87–41.84)	0.077	0.359
Visit to a homeopathic practitioner	Expectancy	146/2,868	0.95 (0.67–1.34)	0.93 (0.66–1.32)	0.686	0.873
Chiropractic care	Use	167/3,022	2.17 (1.48–3.18)	2.16 (1.46–3.19)	<0.001	<0.001
Chiropractic care †	Favorability	37/368	1.21 (0.35–4.17)	0.93 (0.32–3.63)	0.907	0.988
Chiropractic care	Expectancy	130/2,654	0.85 (0.60–1.22)	0.84 (0.59–1.21)	0.355	0.663
Osteopathic manipulation	Use	167/3,022	3.14 (1.77–5.56)	3.17 (1.74–5.76)	<0.001	<0.001
Osteopathic manipulation †	Favorability	15/102	2.48 (0.52–11.80)	3.22 (0.73–23.74)	0.131	0.522
Osteopathic manipulation	Expectancy	152/2,920	0.85 (0.61–1.18)	0.84 (0.60–1.18)	0.322	0.663
Massage therapy	Use	167/3,022	2.12 (1.49–3.02)	2.23 (1.54–3.22)	<0.001	<0.001

Approach/composite	Outcome	CFS n/total	Crude OR (95% CI)	Adjusted OR (95% CI)	p	q
Massage therapy †	Favorability	47/492	2.04 (0.48–8.75)	1.60 (0.49–8.15)	0.468	0.742
Massage therapy	Expectancy	120/2,530	0.92 (0.63–1.35)	0.94 (0.63–1.39)	0.742	0.873
Light, magnetic, or electrical approaches other than physical therapy	Use	167/3,022	2.57 (1.66–3.98)	2.60 (1.66–4.06)	<0.001	<0.001
Light, magnetic, or electrical approaches other than physical therapy	Favorability	27/226	0.77 (0.29–2.05)	0.64 (0.19–2.16)	0.468	0.742
Light, magnetic, or electrical approaches other than physical therapy	Expectancy	140/2,796	1.01 (0.72–1.43)	1.01 (0.71–1.44)	0.941	0.980
Other nonconventional devices	Use	167/3,022	2.03 (1.20–3.44)	2.17 (1.27–3.73)	0.005	0.012
Other nonconventional devices	Favorability	17/168	0.67 (0.22–2.04)	0.36 (0.07–1.96)	0.236	0.661
Other nonconventional devices	Expectancy	150/2,854	1.21 (0.86–1.68)	1.20 (0.86–1.69)	0.284	0.663
Spiritual healing	Use	167/3,022	1.69 (1.00–2.86)	1.73 (1.01–2.98)	0.0472	0.066
Spiritual healing †	Favorability	17/196	1.09 (0.30–4.02)	1.63 (0.45–7.66)	0.477	0.742
Spiritual healing	Expectancy	150/2,826	1.16 (0.83–1.63)	1.18 (0.84–1.66)	0.350	0.663
Prayer	Use	167/3,022	1.39 (0.99–1.96)	1.31 (0.92–1.87)	0.135	0.151
Prayer	Favorability	119/1,948	0.58 (0.31–1.08)	0.55 (0.29–1.05)	0.070	0.359
Prayer	Expectancy	48/1,074	0.62 (0.32–1.21)	0.64 (0.32–1.27)	0.201	0.663
Mindfulness or spiritual practices other than prayer	Use	167/3,022	1.52 (1.11–2.08)	1.53 (1.11–2.11)	0.009	0.019
Mindfulness or spiritual practices other than prayer	Favorability	77/1,106	0.39 (0.21–0.73)	0.37 (0.19–0.70)	0.002	0.068
Mindfulness or spiritual practices other than prayer	Expectancy	90/1,916	0.87 (0.56–1.34)	0.88 (0.56–1.36)	0.553	0.873
Shamanic journey	Use	167/3,022	1.29 (0.68–2.43)	1.27 (0.66–2.44)	0.470	0.470
Shamanic journey †	Favorability	11/159	NE	8.24 (0.98–1072.78)	0.0526	0.359
Shamanic journey	Expectancy	156/2,863	0.99 (0.68–1.42)	1.00 (0.68–1.45)	0.980	0.980
Guided hypnotherapy	Use	167/3,022	2.11 (1.14–3.92)	2.17 (1.13–4.16)	0.020	0.034
Guided hypnotherapy †	Favorability	12/113	0.62 (0.17–2.25)	0.56 (0.15–2.33)	0.416	0.742
Guided hypnotherapy	Expectancy	155/2,909	1.19 (0.85–1.66)	1.18 (0.84–1.66)	0.327	0.663
Self-hypnosis	Use	167/3,022	1.32 (0.74–2.38)	1.40 (0.77–2.56)	0.271	0.281
Self-hypnosis †	Favorability	13/184	1.62 (0.35–7.64)	2.11 (0.51–12.33)	0.316	0.738
Self-hypnosis	Expectancy	154/2,838	1.04 (0.73–1.50)	1.03 (0.71–1.49)	0.875	0.980
Deep breathing exercises	Use	167/3,022	1.57 (1.14–2.16)	1.51 (1.09–2.10)	0.013	0.024
Deep breathing exercises †	Favorability	100/1,491	1.72 (0.62–4.78)	1.48 (0.62–4.57)	0.412	0.742
Deep breathing exercises	Expectancy	67/1,531	0.69 (0.42–1.12)	0.73 (0.44–1.21)	0.219	0.663
Progressive relaxation	Use	167/3,022	1.87 (1.35–2.59)	1.95 (1.39–2.72)	<0.001	<0.001
Progressive relaxation	Favorability	62/748	1.03 (0.40–2.68)	1.00 (0.37–2.69)	0.998	0.998
Progressive relaxation	Expectancy	105/2,274	0.92 (0.62–1.36)	0.92 (0.62–1.38)	0.693	0.873
Meditation	Use	167/3,022	1.41 (1.04–1.93)	1.42 (1.03–1.96)	0.031	0.0462
Meditation	Favorability	83/1,257	1.04 (0.44–2.45)	0.91 (0.38–2.19)	0.831	0.988
Meditation	Expectancy	84/1,765	0.75 (0.48–1.16)	0.79 (0.50–1.23)	0.295	0.663
Biofeedback	Use	167/3,022	1.62 (0.86–3.07)	1.66 (0.86–3.20)	0.133	0.151
Biofeedback †	Favorability	11/130	1.20 (0.24–5.89)	0.92 (0.19–5.97)	0.921	0.988
Biofeedback	Expectancy	156/2,892	1.17 (0.84–1.65)	1.19 (0.85–1.68)	0.309	0.663
Guided imagery	Use	167/3,022	1.46 (0.88–2.43)	1.50 (0.89–2.51)	0.129	0.151
Guided imagery †	Favorability	18/236	1.18 (0.26–5.40)	1.43 (0.35–8.35)	0.638	0.850
Guided imagery	Expectancy	149/2,786	0.96 (0.67–1.38)	0.94 (0.65–1.36)	0.747	0.873
Acupuncture	Use	167/3,022	2.44 (1.52–3.93)	2.69 (1.64–4.42)	<0.001	<0.001

Approach/composite	Outcome	CFS n/total	Crude OR (95% CI)	Adjusted OR (95% CI)	p	q
Acupuncture	Favorability	22/189	0.63 (0.24–1.66)	0.72 (0.24–2.16)	0.555	0.777
Acupuncture	Expectancy	145/2,833	0.92 (0.66–1.29)	0.94 (0.67–1.33)	0.748	0.873
Yoga	Use	167/3,022	1.29 (0.89–1.86)	1.31 (0.89–1.94)	0.176	0.190
Yoga	Favorability	40/602	0.37 (0.15–0.89)	0.35 (0.14–0.90)	0.030	0.277
Yoga	Expectancy	127/2,420	0.91 (0.64–1.30)	0.94 (0.65–1.35)	0.731	0.873
Tai chi or qigong	Use	167/3,022	1.50 (0.81–2.77)	1.66 (0.89–3.09)	0.112	0.142
Tai chi or qigong †	Favorability	12/152	0.44 (0.11–1.79)	0.60 (0.14–2.91)	0.503	0.742
Tai chi or qigong	Expectancy	155/2,870	0.87 (0.63–1.22)	0.89 (0.64–1.25)	0.501	0.873
Movement therapy	Use	167/3,022	1.69 (1.07–2.67)	1.70 (1.06–2.71)	0.026	0.043
Movement therapy †	Favorability	23/270	0.78 (0.25–2.44)	0.91 (0.30–3.32)	0.880	0.988
Movement therapy	Expectancy	144/2,752	0.90 (0.64–1.25)	0.94 (0.67–1.33)	0.743	0.873
Art, music, or dance	Use	167/3,022	1.86 (1.34–2.57)	1.82 (1.30–2.55)	<0.001	0.002
Art, music, or dance †	Favorability	108/1,526	2.63 (0.64–10.88)	1.84 (0.62–9.01)	0.308	0.738
Art, music, or dance	Expectancy	59/1,496	0.75 (0.45–1.28)	0.75 (0.44–1.28)	0.291	0.663
Traditional healing systems	Use	167/3,022	1.37 (0.86–2.19)	1.48 (0.91–2.38)	0.111	0.142
Traditional healing systems †	Favorability	22/306	1.16 (0.33–4.09)	1.04 (0.33–4.28)	0.952	0.988
Traditional healing systems	Expectancy	145/2,716	0.74 (0.53–1.05)	0.75 (0.52–1.06)	0.103	0.663
Visit to a naturopathic physician	Use	167/3,022	2.90 (1.78–4.73)	3.13 (1.87–5.22)	<0.001	<0.001
Visit to a naturopathic physician †	Favorability	21/156	8.12 (1.05–62.65)	5.88 (1.17–60.44)	0.030	0.277
Visit to a naturopathic physician	Expectancy	146/2,866	1.01 (0.72–1.41)	1.01 (0.71–1.42)	0.959	0.980
Heat or cold therapy	Use	167/3,022	2.23 (1.63–3.05)	2.18 (1.58–3.00)	<0.001	<0.001
Heat or cold therapy	Favorability	90/1,073	0.81 (0.38–1.74)	0.78 (0.35–1.73)	0.540	0.616
Heat or cold therapy	Expectancy	77/1,949	0.90 (0.57–1.43)	0.95 (0.59–1.51)	0.815	0.815
Over-the-counter medication	Use	167/3,022	1.80 (1.09–2.96)	1.67 (1.00–2.78)	0.0496	0.0496
Over-the-counter medication	Favorability	149/2,495	0.50 (0.33–0.75)	0.51 (0.33–0.77)	0.001	0.010
Over-the-counter medication	Expectancy	18/527	0.74 (0.29–1.92)	0.64 (0.24–1.73)	0.380	0.686
Physical therapy	Use	167/3,022	2.23 (1.61–3.10)	2.22 (1.59–3.10)	<0.001	<0.001
Physical therapy	Favorability	61/646	0.85 (0.43–1.69)	0.83 (0.40–1.70)	0.606	0.616
Physical therapy	Expectancy	106/2,376	0.79 (0.53–1.18)	0.84 (0.55–1.26)	0.392	0.686
Psychotherapy	Use	167/3,022	2.25 (1.52–3.33)	2.22 (1.48–3.33)	<0.001	<0.001
Psychotherapy †	Favorability	35/336	3.53 (0.82–15.15)	3.21 (0.97–16.66)	0.057	0.199
Psychotherapy	Expectancy	132/2,686	1.09 (0.76–1.55)	1.12 (0.78–1.60)	0.556	0.779
Prescription diet	Use	167/3,022	2.90 (2.03–4.12)	2.88 (2.00–4.15)	<0.001	<0.001
Prescription diet	Favorability	48/397	1.44 (0.62–3.36)	2.21 (0.89–5.45)	0.087	0.202
Prescription diet	Expectancy	119/2,625	0.80 (0.55–1.16)	0.82 (0.56–1.21)	0.316	0.686
Prescription medication	Use	167/3,022	3.44 (2.07–5.72)	3.46 (2.05–5.84)	<0.001	<0.001
Prescription medication	Favorability	150/2,204	1.13 (0.69–1.86)	1.18 (0.71–1.95)	0.525	0.616
Prescription medication	Expectancy	17/818	0.37 (0.14–0.98)	0.29 (0.11–0.79)	0.015	0.107
Surgery	Use	167/3,022	2.04 (1.42–2.94)	2.04 (1.41–2.96)	<0.001	<0.001
Surgery	Favorability	42/446	1.03 (0.46–2.32)	1.25 (0.52–3.01)	0.616	0.616
Surgery	Expectancy	125/2,576	1.00 (0.69–1.44)	1.06 (0.73–1.54)	0.761	0.815

Table S1A. Recorded modality labels and analytic classification.

The category assignments are analytic classifications retained from the parent survey. Conventional approaches do not contribute to CHA composites.

Recorded item label	Classification
Vitamin or mineral supplements	Nutritional
Other Dietary supplements or natural products that are not prescription medications	Nutritional
Visit to an herbalist	Nutritional
Special diet not prescribed by a doctor	Nutritional
Visit to a homeopathic practitioner	Nutritional
Visit to a chiropractor	Physical
Osteopathic manipulation	Physical
Massage therapy	Physical
Light, magnetic, or electrical stimulation other than conventional physical therapy	Physical
Other non-conventional devices	Physical
Visit to a spiritual healer	Psychological
Prayer	Psychological
Mindfulness or spiritual practices other than prayer	Psychological
Shamanic journeys	Psychological
Hypnosis with a therapist	Psychological
Self-hypnosis	Psychological
Deep breathing exercises	Combined
Progressive relaxation	Combined
Meditation	Combined
Biofeedback	Combined
Guided imagery	Combined
Visit to an acupuncturist	Combined
Yoga	Combined
Tai chi or qi gong	Combined
Movement therapies other than yoga, tai chi, or qi gong	Combined
Art, music, or dance	Combined
Traditional healing systems such as Traditional Chinese medicine, Ayurveda, or from other cultural traditions	Systems
Visit to a naturopathic physician	Systems
Heat or cold therapies	Conventional comparator
Over-the-counter, non-prescription medications	Conventional comparator
Physical therapy	Conventional comparator
Psychotherapy	Conventional comparator
Special diet prescribed by a doctor	Conventional comparator
Prescription medications	Conventional comparator
Surgery	Conventional comparator

Table S1B. Historical composite-cutoff estimates with historical pooled and revised question-based multiplicity corrections.

These estimates retain the historical composite definition ≥3.5. Historical q pools 102 CHA or 21 conventional tests. Question-based q uses 6 overall/category CHA, 28 individual CHA, or 7 conventional tests, separately for each outcome. Comparing the two q columns isolates the family change while retaining the old cutoff. The conventional pooled correction was itself first added during revision.

Approach/composite	Outcome	Historical aOR (95% CI)	p	Historical pooled q	Question-based q
Overall CHA	Use	2.46 (0.97–8.91)	0.061	0.206	0.065
Overall CHA	Favorability	0.71 (0.39–1.29)	0.258	0.496	0.515
Overall CHA	Expectancy	1.10 (0.79–1.52)	0.580	0.731	0.696
Nutritional	Use	2.25 (1.34–3.80)	0.002	0.019	0.004
Nutritional	Favorability	1.28 (0.70–2.32)	0.423	0.625	0.568
Nutritional	Expectancy	0.82 (0.59–1.14)	0.242	0.474	0.483
Physical	Use	2.22 (1.60–3.07)	<0.001	<0.001	<0.001
Physical	Favorability	1.26 (0.54–2.92)	0.588	0.732	0.588
Physical	Expectancy	0.91 (0.66–1.26)	0.574	0.731	0.696
Psychological	Use	1.44 (0.98–2.13)	0.065	0.213	0.065
Psychological	Favorability	0.53 (0.30–0.94)	0.029	0.117	0.176
Psychological	Expectancy	1.05 (0.74–1.49)	0.781	0.866	0.781
Combined	Use	1.89 (1.24–2.88)	0.003	0.021	0.004
Combined	Favorability	0.78 (0.39–1.54)	0.473	0.658	0.568
Combined	Expectancy	0.81 (0.58–1.13)	0.225	0.468	0.483
Systems	Use	1.99 (1.32–3.01)	0.001	0.010	0.003
Systems	Favorability	2.51 (0.78–12.76)	0.134	0.327	0.403
Systems	Expectancy	0.80 (0.57–1.11)	0.181	0.421	0.483
Vitamin or mineral supplements	Use	1.77 (1.15–2.73)	0.010	0.0536	0.020
Vitamin or mineral supplements	Favorability	1.11 (0.58–2.13)	0.747	0.838	0.951
Vitamin or mineral supplements	Expectancy	0.61 (0.27–1.37)	0.232	0.472	0.663
Other dietary supplements or natural products	Use	1.59 (1.15–2.19)	0.005	0.030	0.012
Other dietary supplements or natural products	Favorability	1.60 (0.79–3.23)	0.193	0.435	0.624
Other dietary supplements or natural products	Expectancy	0.77 (0.48–1.23)	0.276	0.508	0.663
Herbalist consultation	Use	2.11 (1.27–3.51)	0.004	0.027	0.011
Herbalist consultation	Favorability	2.34 (0.67–12.69)	0.200	0.435	0.624
Herbalist consultation	Expectancy	0.83 (0.58–1.17)	0.279	0.508	0.663
Self-directed special diet	Use	1.54 (1.05–2.27)	0.028	0.117	0.043
Self-directed special diet	Favorability	1.39 (0.53–3.64)	0.498	0.667	0.742
Self-directed special diet	Expectancy	0.75 (0.52–1.08)	0.118	0.325	0.663
Visit to a homeopathic practitioner	Use	3.25 (1.96–5.40)	<0.001	<0.001	<0.001
Visit to a homeopathic practitioner	Favorability	4.19 (0.87–41.84)	0.077	0.238	0.359
Visit to a homeopathic practitioner	Expectancy	0.93 (0.66–1.32)	0.686	0.831	0.873
Chiropractic care	Use	2.16 (1.46–3.19)	<0.001	0.001	<0.001
Chiropractic care	Favorability	0.93 (0.32–3.63)	0.907	0.964	0.988
Chiropractic care	Expectancy	0.84 (0.59–1.21)	0.355	0.549	0.663
Osteopathic manipulation	Use	3.17 (1.74–5.76)	<0.001	0.002	<0.001
Osteopathic manipulation	Favorability	3.22 (0.73–23.74)	0.131	0.327	0.522
Osteopathic manipulation	Expectancy	0.84 (0.60–1.18)	0.322	0.521	0.663
Massage therapy	Use	2.23 (1.54–3.22)	<0.001	<0.001	<0.001

Approach/composite	Outcome	Historical aOR (95% CI)	p	Historical pooled q	Question-based q
Massage therapy	Favorability	1.60 (0.49–8.15)	0.468	0.658	0.742
Massage therapy	Expectancy	0.94 (0.63–1.39)	0.742	0.838	0.873
Light, magnetic, or electrical approaches other than physical therapy	Use	2.60 (1.66–4.06)	<0.001	<0.001	<0.001
Light, magnetic, or electrical approaches other than physical therapy	Favorability	0.64 (0.19–2.16)	0.468	0.658	0.742
Light, magnetic, or electrical approaches other than physical therapy	Expectancy	1.01 (0.71–1.44)	0.941	0.979	0.980
Other nonconventional devices	Use	2.17 (1.27–3.73)	0.005	0.030	0.012
Other nonconventional devices	Favorability	0.36 (0.07–1.96)	0.236	0.472	0.661
Other nonconventional devices	Expectancy	1.20 (0.86–1.69)	0.284	0.508	0.663
Spiritual healing	Use	1.73 (1.01–2.98)	0.0472	0.172	0.066
Spiritual healing	Favorability	1.63 (0.45–7.66)	0.477	0.658	0.742
Spiritual healing	Expectancy	1.18 (0.84–1.66)	0.350	0.549	0.663
Prayer	Use	1.31 (0.92–1.87)	0.135	0.327	0.151
Prayer	Favorability	0.55 (0.29–1.05)	0.070	0.222	0.359
Prayer	Expectancy	0.64 (0.32–1.27)	0.201	0.435	0.663
Mindfulness or spiritual practices other than prayer	Use	1.53 (1.11–2.11)	0.009	0.0511	0.019
Mindfulness or spiritual practices other than prayer	Favorability	0.37 (0.19–0.70)	0.002	0.019	0.068
Mindfulness or spiritual practices other than prayer	Expectancy	0.88 (0.56–1.36)	0.553	0.717	0.873
Shamanic journey	Use	1.27 (0.66–2.44)	0.470	0.658	0.470
Shamanic journey	Favorability	8.24 (0.98–1072.78)	0.0526	0.185	0.359
Shamanic journey	Expectancy	1.00 (0.68–1.45)	0.980	0.989	0.980
Guided hypnotherapy	Use	2.17 (1.13–4.16)	0.020	0.095	0.034
Guided hypnotherapy	Favorability	0.56 (0.15–2.33)	0.416	0.623	0.742
Guided hypnotherapy	Expectancy	1.18 (0.84–1.66)	0.327	0.521	0.663
Self-hypnosis	Use	1.40 (0.77–2.56)	0.271	0.508	0.281
Self-hypnosis	Favorability	2.11 (0.51–12.33)	0.316	0.520	0.738
Self-hypnosis	Expectancy	1.03 (0.71–1.49)	0.875	0.944	0.980
Deep breathing exercises	Use	1.51 (1.09–2.10)	0.013	0.067	0.024
Deep breathing exercises	Favorability	1.48 (0.62–4.57)	0.412	0.623	0.742
Deep breathing exercises	Expectancy	0.73 (0.44–1.21)	0.219	0.466	0.663
Progressive relaxation	Use	1.95 (1.39–2.72)	<0.001	0.001	<0.001
Progressive relaxation	Favorability	1.00 (0.37–2.69)	0.998	0.998	0.998
Progressive relaxation	Expectancy	0.92 (0.62–1.38)	0.693	0.831	0.873
Meditation	Use	1.42 (1.03–1.96)	0.031	0.118	0.0462
Meditation	Favorability	0.91 (0.38–2.19)	0.831	0.912	0.988
Meditation	Expectancy	0.79 (0.50–1.23)	0.295	0.509	0.663
Biofeedback	Use	1.66 (0.86–3.20)	0.133	0.327	0.151
Biofeedback	Favorability	0.92 (0.19–5.97)	0.921	0.969	0.988
Biofeedback	Expectancy	1.19 (0.85–1.68)	0.309	0.517	0.663
Guided imagery	Use	1.50 (0.89–2.51)	0.129	0.327	0.151
Guided imagery	Favorability	1.43 (0.35–8.35)	0.638	0.784	0.850
Guided imagery	Expectancy	0.94 (0.65–1.36)	0.747	0.838	0.873
Acupuncture	Use	2.69 (1.64–4.42)	<0.001	0.001	<0.001
Acupuncture	Favorability	0.72 (0.24–2.16)	0.555	0.717	0.777

Approach/composite	Outcome	Historical aOR (95% CI)	p	Historical pooled q	Question-based q
Acupuncture	Expectancy	0.94 (0.67–1.33)	0.748	0.838	0.873
Yoga	Use	1.31 (0.89–1.94)	0.176	0.418	0.190
Yoga	Favorability	0.35 (0.14–0.90)	0.030	0.117	0.277
Yoga	Expectancy	0.94 (0.65–1.35)	0.731	0.838	0.873
Tai chi or qigong	Use	1.66 (0.89–3.09)	0.112	0.316	0.142
Tai chi or qigong	Favorability	0.60 (0.14–2.91)	0.503	0.667	0.742
Tai chi or qigong	Expectancy	0.89 (0.64–1.25)	0.501	0.667	0.873
Movement therapy	Use	1.70 (1.06–2.71)	0.026	0.117	0.043
Movement therapy	Favorability	0.91 (0.30–3.32)	0.880	0.944	0.988
Movement therapy	Expectancy	0.94 (0.67–1.33)	0.743	0.838	0.873
Art, music, or dance	Use	1.82 (1.30–2.55)	<0.001	0.005	0.002
Art, music, or dance	Favorability	1.84 (0.62–9.01)	0.308	0.517	0.738
Art, music, or dance	Expectancy	0.75 (0.44–1.28)	0.291	0.509	0.663
Traditional healing systems	Use	1.48 (0.91–2.38)	0.111	0.316	0.142
Traditional healing systems	Favorability	1.04 (0.33–4.28)	0.952	0.979	0.988
Traditional healing systems	Expectancy	0.75 (0.52–1.06)	0.103	0.308	0.663
Visit to a naturopathic physician	Use	3.13 (1.87–5.22)	<0.001	<0.001	<0.001
Visit to a naturopathic physician	Favorability	5.88 (1.17–60.44)	0.030	0.117	0.277
Visit to a naturopathic physician	Expectancy	1.01 (0.71–1.42)	0.959	0.979	0.980
Heat or cold therapy	Use	2.18 (1.58–3.00)	<0.001	<0.001	<0.001
Heat or cold therapy	Favorability	0.78 (0.35–1.73)	0.540	0.681	0.616
Heat or cold therapy	Expectancy	0.95 (0.59–1.51)	0.815	0.815	0.815
Over-the-counter medication	Use	1.67 (1.00–2.78)	0.0496	0.116	0.0496
Over-the-counter medication	Favorability	0.51 (0.33–0.77)	0.001	0.004	0.010
Over-the-counter medication	Expectancy	0.64 (0.24–1.73)	0.380	0.588	0.686
Physical therapy	Use	2.22 (1.59–3.10)	<0.001	<0.001	<0.001
Physical therapy	Favorability	0.83 (0.40–1.70)	0.606	0.681	0.616
Physical therapy	Expectancy	0.84 (0.55–1.26)	0.392	0.588	0.686
Psychotherapy	Use	2.22 (1.48–3.33)	<0.001	<0.001	<0.001
Psychotherapy	Favorability	3.21 (0.97–16.66)	0.057	0.120	0.199
Psychotherapy	Expectancy	1.12 (0.78–1.60)	0.556	0.681	0.779
Prescription diet	Use	2.88 (2.00–4.15)	<0.001	<0.001	<0.001
Prescription diet	Favorability	2.21 (0.89–5.45)	0.087	0.165	0.202
Prescription diet	Expectancy	0.82 (0.56–1.21)	0.316	0.554	0.686
Prescription medication	Use	3.46 (2.05–5.84)	<0.001	<0.001	<0.001
Prescription medication	Favorability	1.18 (0.71–1.95)	0.525	0.681	0.616
Prescription medication	Expectancy	0.29 (0.11–0.79)	0.015	0.040	0.107
Surgery	Use	2.04 (1.41–2.96)	<0.001	<0.001	<0.001
Surgery	Favorability	1.25 (0.52–3.01)	0.616	0.681	0.616
Surgery	Expectancy	1.06 (0.73–1.54)	0.761	0.799	0.815

Table S1C. Comparison of inference under historical correction, changed testing families alone, and the final revised analysis.

All 123 contrasts are included. Family-only q changes the correction families while retaining historical ≥3.5 composite coding. Final q also incorporates the revised >3.5 composite definition. Changes were made during revision after earlier results were known. Table S1B supplies the historical CIs; Table S1 supplies the revised CIs.

Approach/composite	Outcome	Historical aOR	Revised aOR	Historical q	Family-only q	Final q
Overall CHA	Use	2.46	2.46	0.206	0.065	0.065
Overall CHA	Favorability	0.71	0.67	0.496	0.515	0.216
Overall CHA	Expectancy	1.10	1.06	0.731	0.696	0.847
Nutritional	Use	2.25	2.25	0.019	0.004	0.004
Nutritional	Favorability	1.28	1.16	0.625	0.568	0.579
Nutritional	Expectancy	0.82	0.76	0.474	0.483	0.694
Physical	Use	2.22	2.22	<0.001	<0.001	<0.001
Physical	Favorability	1.26	1.57	0.732	0.588	0.344
Physical	Expectancy	0.91	0.97	0.731	0.696	0.847
Psychological	Use	1.44	1.44	0.213	0.065	0.065
Psychological	Favorability	0.53	0.46	0.117	0.176	0.013
Psychological	Expectancy	1.05	1.10	0.866	0.781	0.847
Combined	Use	1.89	1.89	0.021	0.004	0.004
Combined	Favorability	0.78	0.64	0.658	0.568	0.216
Combined	Expectancy	0.81	0.88	0.468	0.483	0.847
Systems	Use	1.99	1.99	0.010	0.003	0.003
Systems	Favorability	2.51	2.93	0.327	0.403	0.216
Systems	Expectancy	0.80	0.93	0.421	0.483	0.847
Vitamin or mineral supplements	Use	1.77	1.77	0.0536	0.020	0.020
Vitamin or mineral supplements	Favorability	1.11	1.11	0.838	0.951	0.951
Vitamin or mineral supplements	Expectancy	0.61	0.61	0.472	0.663	0.663
Other dietary supplements or natural products	Use	1.59	1.59	0.030	0.012	0.012
Other dietary supplements or natural products	Favorability	1.60	1.60	0.435	0.624	0.624
Other dietary supplements or natural products	Expectancy	0.77	0.77	0.508	0.663	0.663
Herbalist consultation	Use	2.11	2.11	0.027	0.011	0.011
Herbalist consultation	Favorability	2.34	2.34	0.435	0.624	0.624
Herbalist consultation	Expectancy	0.83	0.83	0.508	0.663	0.663
Self-directed special diet	Use	1.54	1.54	0.117	0.043	0.043
Self-directed special diet	Favorability	1.39	1.39	0.667	0.742	0.742
Self-directed special diet	Expectancy	0.75	0.75	0.325	0.663	0.663
Visit to a homeopathic practitioner	Use	3.25	3.25	<0.001	<0.001	<0.001
Visit to a homeopathic practitioner	Favorability	4.19	4.19	0.238	0.359	0.359
Visit to a homeopathic practitioner	Expectancy	0.93	0.93	0.831	0.873	0.873
Chiropractic care	Use	2.16	2.16	0.001	<0.001	<0.001
Chiropractic care	Favorability	0.93	0.93	0.964	0.988	0.988
Chiropractic care	Expectancy	0.84	0.84	0.549	0.663	0.663
Osteopathic manipulation	Use	3.17	3.17	0.002	<0.001	<0.001
Osteopathic manipulation	Favorability	3.22	3.22	0.327	0.522	0.522
Osteopathic manipulation	Expectancy	0.84	0.84	0.521	0.663	0.663
Massage therapy	Use	2.23	2.23	<0.001	<0.001	<0.001

Approach/composite	Outcome	Historical aOR	Revised aOR	Historical q	Family-only q	Final q
Massage therapy	Favorability	1.60	1.60	0.658	0.742	0.742
Massage therapy	Expectancy	0.94	0.94	0.838	0.873	0.873
Light, magnetic, or electrical approaches other than physical therapy	Use	2.60	2.60	<0.001	<0.001	<0.001
Light, magnetic, or electrical approaches other than physical therapy	Favorability	0.64	0.64	0.658	0.742	0.742
Light, magnetic, or electrical approaches other than physical therapy	Expectancy	1.01	1.01	0.979	0.980	0.980
Other nonconventional devices	Use	2.17	2.17	0.030	0.012	0.012
Other nonconventional devices	Favorability	0.36	0.36	0.472	0.661	0.661
Other nonconventional devices	Expectancy	1.20	1.20	0.508	0.663	0.663
Spiritual healing	Use	1.73	1.73	0.172	0.066	0.066
Spiritual healing	Favorability	1.63	1.63	0.658	0.742	0.742
Spiritual healing	Expectancy	1.18	1.18	0.549	0.663	0.663
Prayer	Use	1.31	1.31	0.327	0.151	0.151
Prayer	Favorability	0.55	0.55	0.222	0.359	0.359
Prayer	Expectancy	0.64	0.64	0.435	0.663	0.663
Mindfulness or spiritual practices other than prayer	Use	1.53	1.53	0.0511	0.019	0.019
Mindfulness or spiritual practices other than prayer	Favorability	0.37	0.37	0.019	0.068	0.068
Mindfulness or spiritual practices other than prayer	Expectancy	0.88	0.88	0.717	0.873	0.873
Shamanic journey	Use	1.27	1.27	0.658	0.470	0.470
Shamanic journey	Favorability	8.24	8.24	0.185	0.359	0.359
Shamanic journey	Expectancy	1.00	1.00	0.989	0.980	0.980
Guided hypnotherapy	Use	2.17	2.17	0.095	0.034	0.034
Guided hypnotherapy	Favorability	0.56	0.56	0.623	0.742	0.742
Guided hypnotherapy	Expectancy	1.18	1.18	0.521	0.663	0.663
Self-hypnosis	Use	1.40	1.40	0.508	0.281	0.281
Self-hypnosis	Favorability	2.11	2.11	0.520	0.738	0.738
Self-hypnosis	Expectancy	1.03	1.03	0.944	0.980	0.980
Deep breathing exercises	Use	1.51	1.51	0.067	0.024	0.024
Deep breathing exercises	Favorability	1.48	1.48	0.623	0.742	0.742
Deep breathing exercises	Expectancy	0.73	0.73	0.466	0.663	0.663
Progressive relaxation	Use	1.95	1.95	0.001	<0.001	<0.001
Progressive relaxation	Favorability	1.00	1.00	0.998	0.998	0.998
Progressive relaxation	Expectancy	0.92	0.92	0.831	0.873	0.873
Meditation	Use	1.42	1.42	0.118	0.0462	0.0462
Meditation	Favorability	0.91	0.91	0.912	0.988	0.988
Meditation	Expectancy	0.79	0.79	0.509	0.663	0.663
Biofeedback	Use	1.66	1.66	0.327	0.151	0.151
Biofeedback	Favorability	0.92	0.92	0.969	0.988	0.988
Biofeedback	Expectancy	1.19	1.19	0.517	0.663	0.663
Guided imagery	Use	1.50	1.50	0.327	0.151	0.151
Guided imagery	Favorability	1.43	1.43	0.784	0.850	0.850
Guided imagery	Expectancy	0.94	0.94	0.838	0.873	0.873
Acupuncture	Use	2.69	2.69	0.001	<0.001	<0.001
Acupuncture	Favorability	0.72	0.72	0.717	0.777	0.777

Approach/composite	Outcome	Historical aOR	Revised aOR	Historical q	Family-only q	Final q
Acupuncture	Expectancy	0.94	0.94	0.838	0.873	0.873
Yoga	Use	1.31	1.31	0.418	0.190	0.190
Yoga	Favorability	0.35	0.35	0.117	0.277	0.277
Yoga	Expectancy	0.94	0.94	0.838	0.873	0.873
Tai chi or qigong	Use	1.66	1.66	0.316	0.142	0.142
Tai chi or qigong	Favorability	0.60	0.60	0.667	0.742	0.742
Tai chi or qigong	Expectancy	0.89	0.89	0.667	0.873	0.873
Movement therapy	Use	1.70	1.70	0.117	0.043	0.043
Movement therapy	Favorability	0.91	0.91	0.944	0.988	0.988
Movement therapy	Expectancy	0.94	0.94	0.838	0.873	0.873
Art, music, or dance	Use	1.82	1.82	0.005	0.002	0.002
Art, music, or dance	Favorability	1.84	1.84	0.517	0.738	0.738
Art, music, or dance	Expectancy	0.75	0.75	0.509	0.663	0.663
Traditional healing systems	Use	1.48	1.48	0.316	0.142	0.142
Traditional healing systems	Favorability	1.04	1.04	0.979	0.988	0.988
Traditional healing systems	Expectancy	0.75	0.75	0.308	0.663	0.663
Visit to a naturopathic physician	Use	3.13	3.13	<0.001	<0.001	<0.001
Visit to a naturopathic physician	Favorability	5.88	5.88	0.117	0.277	0.277
Visit to a naturopathic physician	Expectancy	1.01	1.01	0.979	0.980	0.980
Heat or cold therapy	Use	2.18	2.18	<0.001	<0.001	<0.001
Heat or cold therapy	Favorability	0.78	0.78	0.681	0.616	0.616
Heat or cold therapy	Expectancy	0.95	0.95	0.815	0.815	0.815
Over-the-counter medication	Use	1.67	1.67	0.116	0.0496	0.0496
Over-the-counter medication	Favorability	0.51	0.51	0.004	0.010	0.010
Over-the-counter medication	Expectancy	0.64	0.64	0.588	0.686	0.686
Physical therapy	Use	2.22	2.22	<0.001	<0.001	<0.001
Physical therapy	Favorability	0.83	0.83	0.681	0.616	0.616
Physical therapy	Expectancy	0.84	0.84	0.588	0.686	0.686
Psychotherapy	Use	2.22	2.22	<0.001	<0.001	<0.001
Psychotherapy	Favorability	3.21	3.21	0.120	0.199	0.199
Psychotherapy	Expectancy	1.12	1.12	0.681	0.779	0.779
Prescription diet	Use	2.88	2.88	<0.001	<0.001	<0.001
Prescription diet	Favorability	2.21	2.21	0.165	0.202	0.202
Prescription diet	Expectancy	0.82	0.82	0.554	0.686	0.686
Prescription medication	Use	3.46	3.46	<0.001	<0.001	<0.001
Prescription medication	Favorability	1.18	1.18	0.681	0.616	0.616
Prescription medication	Expectancy	0.29	0.29	0.040	0.107	0.107
Surgery	Use	2.04	2.04	<0.001	<0.001	<0.001
Surgery	Favorability	1.25	1.25	0.681	0.616	0.616
Surgery	Expectancy	1.06	1.06	0.799	0.815	0.815

Table S2. Firth penalized-likelihood estimates and initial maximum-likelihood alternatives for all 20 penalized adjusted models.

Firth intervals are profile penalized-likelihood intervals and p-values are penalized likelihood-ratio tests. The initial maximum-likelihood alternatives are not a gold standard in sparse data. “Small cell” means an exposure-by-outcome cell <5; “wide CI” means initial Wald OR upper/lower confidence-limit ratio >50. NE denotes an estimate failing the numerical reporting screen.

Outcome	Firth trigger	Firth aOR (95% CI)	Firth p	Initial ML aOR (95% CI)
Overall CHA; Use	exposure-by-outcome cell <5	2.46 (0.97–8.91)	0.061	2.86 (0.89–9.15)
Systems; Favorability	exposure-by-outcome cell <5	2.93 (0.91–14.83)	0.074	3.74 (0.83–16.77)
Herbalist consultation; Favorability	exposure-by-outcome cell <5	2.34 (0.67–12.69)	0.200	3.09 (0.61–15.71)
Visit to a homeopathic practitioner; Favorability	exposure-by-outcome cell <5; CI upper/lower ratio >50	4.19 (0.87–41.84)	0.077	7.61 (0.78–74.25)
Chiropractic care; Favorability	exposure-by-outcome cell <5	0.93 (0.32–3.63)	0.907	1.03 (0.28–3.79)
Osteopathic manipulation; Favorability	exposure-by-outcome cell <5; CI upper/lower ratio >50	3.22 (0.73–23.74)	0.131	6.61 (0.81–54.13)
Massage therapy; Favorability	exposure-by-outcome cell <5	1.60 (0.49–8.15)	0.468	1.99 (0.44–9.11)
Spiritual healing; Favorability	exposure-by-outcome cell <5	1.63 (0.45–7.66)	0.477	1.96 (0.40–9.52)
Shamanic journey; Favorability	exposure-by-outcome cell <5; separation/unstable coefficient; CI upper/lower ratio >50	8.24 (0.98–1072.78)	0.0526	NE
Guided hypnotherapy; Favorability	exposure-by-outcome cell <5	0.56 (0.15–2.33)	0.416	0.49 (0.10–2.41)
Self-hypnosis; Favorability	exposure-by-outcome cell <5	2.11 (0.51–12.33)	0.316	2.71 (0.47–15.66)
Deep breathing exercises; Favorability	exposure-by-outcome cell <5	1.48 (0.62–4.57)	0.412	1.65 (0.58–4.64)
Biofeedback; Favorability	exposure-by-outcome cell <5; CI upper/lower ratio >50	0.92 (0.19–5.97)	0.921	1.00 (0.14–7.15)
Guided imagery; Favorability	exposure-by-outcome cell <5	1.43 (0.35–8.35)	0.638	1.72 (0.30–9.91)
Tai chi or qigong; Favorability	exposure-by-outcome cell <5	0.60 (0.14–2.91)	0.503	0.52 (0.09–2.98)
Movement therapy; Favorability	exposure-by-outcome cell <5	0.91 (0.30–3.32)	0.880	0.98 (0.27–3.53)
Art, music, or dance; Favorability	exposure-by-outcome cell <5	1.84 (0.62–9.01)	0.308	2.29 (0.54–9.60)
Traditional healing systems; Favorability	exposure-by-outcome cell <5	1.04 (0.33–4.28)	0.952	1.15 (0.29–4.56)
Visit to a naturopathic physician; Favorability	exposure-by-outcome cell <5; CI upper/lower ratio >50	5.88 (1.17–60.44)	0.030	11.10 (1.11–111.11)
Psychotherapy; Favorability	exposure-by-outcome cell <5	3.21 (0.97–16.66)	0.057	4.26 (0.91–20.06)

Table S3. Six-level individual-rating distributions by reported CFS diagnosis and outcome.

Counts retain all six ordered individual-rating levels. CFS 1 denotes a reported clinician diagnosis; CFS 0 denotes no reported diagnosis. Favorability includes modality users and expectancy includes modality nonusers. N is the row total. Composite means are not forced into six response categories.

Approach	Outcome	CFS	N	1	2	3	4	5	6
Vitamin or mineral supplements	Favorability	0	2,148	39	58	73	576	696	706
Vitamin or mineral supplements	Favorability	1	140	3	0	8	40	50	39
Vitamin or mineral supplements	Expectancy	0	707	109	59	88	265	120	66
Vitamin or mineral supplements	Expectancy	1	27	6	4	3	9	3	2
Other dietary supplements or natural products	Favorability	0	1,247	42	60	74	357	397	317
Other dietary supplements or natural products	Favorability	1	91	2	1	7	33	25	23
Other dietary supplements or natural products	Expectancy	0	1,608	251	159	211	536	316	135
Other dietary supplements or natural products	Expectancy	1	76	16	9	10	26	8	7
Herbalist consultation	Favorability	0	181	7	12	20	41	54	47
Herbalist consultation	Favorability	1	20	0	0	2	5	3	10
Herbalist consultation	Expectancy	0	2,674	786	338	375	713	288	174
Herbalist consultation	Expectancy	1	147	45	23	22	36	15	6
Self-directed special diet	Favorability	0	447	27	24	35	106	132	123
Self-directed special diet	Favorability	1	37	2	0	4	11	10	10
Self-directed special diet	Expectancy	0	2,408	499	282	369	738	349	171
Self-directed special diet	Expectancy	1	130	29	21	22	33	17	8
Visit to a homeopathic practitioner	Favorability	0	133	8	10	13	35	36	31
Visit to a homeopathic practitioner	Favorability	1	21	0	0	1	2	11	7
Visit to a homeopathic practitioner	Expectancy	0	2,722	848	350	447	653	290	134
Visit to a homeopathic practitioner	Expectancy	1	146	51	16	23	35	15	6
Chiropractic care	Favorability	0	331	11	9	12	57	86	156
Chiropractic care	Favorability	1	37	0	1	2	7	11	16
Chiropractic care	Expectancy	0	2,524	595	191	243	691	485	319
Chiropractic care	Expectancy	1	130	33	9	16	28	30	14
Osteopathic manipulation	Favorability	0	87	5	6	13	12	23	28
Osteopathic manipulation	Favorability	1	15	1	1	0	5	1	7
Osteopathic manipulation	Expectancy	0	2,768	782	282	461	715	378	150
Osteopathic manipulation	Expectancy	1	152	43	13	34	27	30	5
Massage therapy	Favorability	0	445	9	11	17	62	111	235
Massage therapy	Favorability	1	47	0	1	1	5	16	24
Massage therapy	Expectancy	0	2,410	483	127	188	709	500	403
Massage therapy	Expectancy	1	120	20	8	14	21	34	23
Light, magnetic, or electrical approaches other than physical therapy	Favorability	0	199	9	10	17	53	60	50
Light, magnetic, or electrical approaches other than physical therapy	Favorability	1	27	1	2	3	4	10	7
Light, magnetic, or electrical approaches other than physical therapy	Expectancy	0	2,656	867	300	417	673	260	139
Light, magnetic, or electrical approaches other than physical therapy	Expectancy	1	140	42	22	19	34	18	5
Other nonconventional devices	Favorability	0	151	7	11	15	33	43	42
Other nonconventional devices	Favorability	1	17	1	1	3	4	5	3
Other nonconventional devices	Expectancy	0	2,704	881	325	484	665	228	121

Approach	Outcome	CFS	N	1	2	3	4	5	6
Other nonconventional devices	Expectancy	1	150	45	12	30	43	12	8
Spiritual healing	Favorability	0	179	10	11	13	38	44	63
Spiritual healing	Favorability	1	17	0	1	2	6	2	6
Spiritual healing	Expectancy	0	2,676	944	374	401	551	227	179
Spiritual healing	Expectancy	1	150	50	18	23	34	13	12
Prayer	Favorability	0	1,829	44	24	43	277	345	1,096
Prayer	Favorability	1	119	4	2	6	26	21	60
Prayer	Expectancy	0	1,026	429	108	130	199	81	79
Prayer	Expectancy	1	48	19	7	10	11	1	0
Mindfulness or spiritual practices other than prayer	Favorability	0	1,029	15	30	37	200	274	473
Mindfulness or spiritual practices other than prayer	Favorability	1	77	3	4	7	21	14	28
Mindfulness or spiritual practices other than prayer	Expectancy	0	1,826	587	224	243	474	192	106
Mindfulness or spiritual practices other than prayer	Expectancy	1	90	29	13	13	25	10	0
Shamanic journey	Favorability	0	148	13	11	20	30	22	52
Shamanic journey	Favorability	1	11	0	0	0	6	2	3
Shamanic journey	Expectancy	0	2,707	1,180	372	437	455	163	100
Shamanic journey	Expectancy	1	156	69	23	23	24	13	4
Guided hypnotherapy	Favorability	0	101	6	5	13	22	19	36
Guided hypnotherapy	Favorability	1	12	2	1	1	2	4	2
Guided hypnotherapy	Expectancy	0	2,754	1,005	351	460	551	259	128
Guided hypnotherapy	Expectancy	1	155	64	12	20	33	19	7
Self-hypnosis	Favorability	0	171	7	13	19	45	31	56
Self-hypnosis	Favorability	1	13	0	0	2	5	0	6
Self-hypnosis	Expectancy	0	2,684	1,150	388	437	430	180	99
Self-hypnosis	Expectancy	1	154	65	23	24	25	13	4
Deep breathing exercises	Favorability	0	1,391	24	30	39	310	421	567
Deep breathing exercises	Favorability	1	100	0	2	2	22	27	47
Deep breathing exercises	Expectancy	0	1,464	360	121	168	489	206	120
Deep breathing exercises	Expectancy	1	67	20	6	10	19	8	4
Progressive relaxation	Favorability	0	686	14	9	34	152	186	291
Progressive relaxation	Favorability	1	62	1	1	3	20	15	22
Progressive relaxation	Expectancy	0	2,169	538	194	275	704	316	142
Progressive relaxation	Expectancy	1	105	32	6	13	27	22	5
Meditation	Favorability	0	1,174	23	20	45	250	351	485
Meditation	Favorability	1	83	1	2	3	20	21	36
Meditation	Expectancy	0	1,681	442	164	193	522	232	128
Meditation	Expectancy	1	84	31	6	9	22	12	4
Biofeedback	Favorability	0	119	10	4	11	31	29	34
Biofeedback	Favorability	1	11	0	1	1	2	4	3
Biofeedback	Expectancy	0	2,736	996	388	485	565	197	105
Biofeedback	Expectancy	1	156	57	20	24	33	17	5
Guided imagery	Favorability	0	218	4	6	18	41	73	76
Guided imagery	Favorability	1	18	0	0	2	4	5	7
Guided imagery	Expectancy	0	2,637	978	383	474	522	174	106
Guided imagery	Expectancy	1	149	55	22	28	29	11	4

Approach	Outcome	CFS	N	1	2	3	4	5	6
Acupuncture	Favorability	0	167	9	15	14	42	31	56
Acupuncture	Favorability	1	22	2	2	3	3	5	7
Acupuncture	Expectancy	0	2,688	814	326	364	708	315	161
Acupuncture	Expectancy	1	145	46	20	18	45	9	7
Yoga	Favorability	0	562	13	6	22	109	150	262
Yoga	Favorability	1	40	2	2	3	7	8	18
Yoga	Expectancy	0	2,293	651	189	263	653	347	190
Yoga	Expectancy	1	127	43	8	13	40	18	5
Tai chi or qigong	Favorability	0	140	3	7	8	30	31	61
Tai chi or qigong	Favorability	1	12	0	3	0	5	0	4
Tai chi or qigong	Expectancy	0	2,715	883	282	393	707	283	167
Tai chi or qigong	Expectancy	1	155	54	19	21	36	19	6
Movement therapy	Favorability	0	247	8	11	16	60	68	84
Movement therapy	Favorability	1	23	1	2	1	8	7	4
Movement therapy	Expectancy	0	2,608	782	239	338	704	350	195
Movement therapy	Expectancy	1	144	48	12	19	40	19	6
Art, music, or dance	Favorability	0	1,418	18	17	32	196	323	832
Art, music, or dance	Favorability	1	108	1	0	1	17	29	60
Art, music, or dance	Expectancy	0	1,437	381	146	201	419	192	98
Art, music, or dance	Expectancy	1	59	21	6	7	10	10	5
Traditional healing systems	Favorability	0	284	9	8	27	91	74	75
Traditional healing systems	Favorability	1	22	0	1	2	7	5	7
Traditional healing systems	Expectancy	0	2,571	720	295	414	728	281	133
Traditional healing systems	Expectancy	1	145	49	17	25	35	15	4
Visit to a naturopathic physician	Favorability	0	135	14	11	14	25	40	31
Visit to a naturopathic physician	Favorability	1	21	0	1	0	10	5	5
Visit to a naturopathic physician	Expectancy	0	2,720	827	354	444	662	279	154
Visit to a naturopathic physician	Expectancy	1	146	47	17	23	37	14	8
Heat or cold therapy	Favorability	0	983	17	20	35	227	352	332
Heat or cold therapy	Favorability	1	90	2	3	3	24	30	28
Heat or cold therapy	Expectancy	0	1,872	369	153	233	649	323	145
Heat or cold therapy	Expectancy	1	77	14	10	9	22	16	6
Over-the-counter medication	Favorability	0	2,346	61	99	132	730	804	520
Over-the-counter medication	Favorability	1	149	4	13	16	51	43	22
Over-the-counter medication	Expectancy	0	509	73	46	71	170	104	45
Over-the-counter medication	Expectancy	1	18	3	2	3	4	3	3
Physical therapy	Favorability	0	585	22	24	46	116	165	212
Physical therapy	Favorability	1	61	3	8	0	12	16	22
Physical therapy	Expectancy	0	2,270	402	126	207	675	540	320
Physical therapy	Expectancy	1	106	22	8	10	29	27	10
Psychotherapy	Favorability	0	301	13	13	27	86	71	91
Psychotherapy	Favorability	1	35	0	0	2	10	9	14
Psychotherapy	Expectancy	0	2,554	804	315	404	601	277	153
Psychotherapy	Expectancy	1	132	40	20	16	36	18	2
Prescription diet	Favorability	0	349	17	23	29	95	98	87

Approach	Outcome	CFS	N	1	2	3	4	5	6
Prescription diet	Favorability	1	48	2	2	3	14	12	15
Prescription diet	Expectancy	0	2,506	427	192	238	824	535	290
Prescription diet	Expectancy	1	119	20	12	15	34	22	16
Prescription medication	Favorability	0	2,054	61	85	143	442	662	661
Prescription medication	Favorability	1	150	4	5	10	37	54	40
Prescription medication	Expectancy	0	801	83	56	61	250	202	149
Prescription medication	Expectancy	1	17	3	2	3	6	2	1
Surgery	Favorability	0	404	23	18	38	66	89	170
Surgery	Favorability	1	42	3	0	5	10	13	11
Surgery	Expectancy	0	2,451	583	177	219	524	486	462
Surgery	Expectancy	1	125	26	18	6	28	22	25

Table S4. Adjusted cumulative-logit models for all 70 six-level individual-rating outcomes.

Unpenalized cumulative-logit models retain the original covariates. OR >1 denotes higher odds of a higher rating under proportional odds. q uses separate 28-test CHA and 7-test conventional families for each rating outcome. All 70 models passed the stated numerical checks; this does not establish proportional odds or eliminate sparse-data bias. CFS n/total is model-specific.

Approach	Outcome	CFS n/total	Ordinal aOR (95% CI)	p	q
Vitamin or mineral supplements	Favorability	140/2,288	0.96 (0.71–1.31)	0.810	0.876
Vitamin or mineral supplements	Expectancy	27/734	0.62 (0.30–1.29)	0.202	0.786
Other dietary supplements or natural products	Favorability	91/1,338	1.08 (0.73–1.59)	0.704	0.876
Other dietary supplements or natural products	Expectancy	76/1,684	0.75 (0.49–1.14)	0.172	0.786
Herbalist consultation	Favorability	20/201	2.74 (1.13–6.65)	0.026	0.241
Herbalist consultation	Expectancy	147/2,821	0.88 (0.65–1.18)	0.394	0.786
Self-directed special diet	Favorability	37/484	1.08 (0.59–1.98)	0.812	0.876
Self-directed special diet	Expectancy	130/2,538	0.83 (0.60–1.13)	0.233	0.786
Visit to a homeopathic practitioner	Favorability	21/154	2.76 (1.08–7.05)	0.034	0.241
Visit to a homeopathic practitioner	Expectancy	146/2,868	0.90 (0.66–1.21)	0.485	0.786
Chiropractic care	Favorability	37/368	0.89 (0.47–1.68)	0.714	0.876
Chiropractic care	Expectancy	130/2,654	0.92 (0.67–1.27)	0.624	0.873
Osteopathic manipulation	Favorability	15/102	2.70 (0.87–8.37)	0.086	0.402
Osteopathic manipulation	Expectancy	152/2,920	1.00 (0.75–1.34)	0.998	0.999
Massage therapy	Favorability	47/492	1.22 (0.68–2.18)	0.505	0.876
Massage therapy	Expectancy	120/2,530	1.22 (0.88–1.70)	0.235	0.786
Light, magnetic, or electrical approaches other than physical therapy	Favorability	27/226	1.32 (0.61–2.83)	0.481	0.876
Light, magnetic, or electrical approaches other than physical therapy	Expectancy	140/2,796	1.03 (0.76–1.39)	0.860	0.999
Other nonconventional devices	Favorability	17/168	0.44 (0.17–1.09)	0.077	0.402
Other nonconventional devices	Expectancy	150/2,854	1.15 (0.86–1.55)	0.346	0.786
Spiritual healing	Favorability	17/196	1.27 (0.47–3.49)	0.637	0.876
Spiritual healing	Expectancy	150/2,826	1.16 (0.86–1.56)	0.342	0.786
Prayer	Favorability	119/1,948	0.65 (0.46–0.93)	0.019	0.241
Prayer	Expectancy	48/1,074	0.83 (0.50–1.38)	0.469	0.786
Mindfulness or spiritual practices other than prayer	Favorability	77/1,106	0.52 (0.33–0.80)	0.003	0.089
Mindfulness or spiritual practices other than prayer	Expectancy	90/1,916	0.86 (0.60–1.26)	0.445	0.786
Shamanic journey	Favorability	11/159	1.12 (0.35–3.60)	0.845	0.876
Shamanic journey	Expectancy	156/2,863	0.99 (0.74–1.34)	0.972	0.999
Guided hypnotherapy	Favorability	12/113	0.39 (0.12–1.29)	0.123	0.492
Guided hypnotherapy	Expectancy	155/2,909	1.00 (0.74–1.35)	0.999	0.999
Self-hypnosis	Favorability	13/184	1.85 (0.59–5.80)	0.291	0.678
Self-hypnosis	Expectancy	154/2,838	1.03 (0.76–1.38)	0.870	0.999
Deep breathing exercises	Favorability	100/1,491	1.31 (0.90–1.92)	0.163	0.507
Deep breathing exercises	Expectancy	67/1,531	0.77 (0.49–1.19)	0.240	0.786
Progressive relaxation	Favorability	62/748	0.75 (0.47–1.22)	0.250	0.636
Progressive relaxation	Expectancy	105/2,274	0.94 (0.65–1.35)	0.731	0.975
Meditation	Favorability	83/1,257	1.06 (0.70–1.62)	0.771	0.876
Meditation	Expectancy	84/1,765	0.73 (0.48–1.10)	0.129	0.786
Biofeedback	Favorability	11/130	1.15 (0.35–3.77)	0.814	0.876
Biofeedback	Expectancy	156/2,892	1.11 (0.83–1.50)	0.474	0.786

Approach	Outcome	CFS n/total	Ordinal aOR (95% CI)	p	q
Guided imagery	Favorability	18/236	1.31 (0.50–3.41)	0.586	0.876
Guided imagery	Expectancy	149/2,786	0.98 (0.73–1.32)	0.901	0.999
Acupuncture	Favorability	22/189	0.99 (0.42–2.30)	0.978	0.978
Acupuncture	Expectancy	145/2,833	0.88 (0.65–1.19)	0.402	0.786
Yoga	Favorability	40/602	0.73 (0.39–1.39)	0.342	0.736
Yoga	Expectancy	127/2,420	0.82 (0.59–1.13)	0.220	0.786
Tai chi or qigong	Favorability	12/152	0.48 (0.14–1.67)	0.247	0.636
Tai chi or qigong	Expectancy	155/2,870	0.91 (0.68–1.22)	0.534	0.786
Movement therapy	Favorability	23/270	0.55 (0.24–1.24)	0.148	0.507
Movement therapy	Expectancy	144/2,752	0.91 (0.67–1.23)	0.532	0.786
Art, music, or dance	Favorability	108/1,526	0.93 (0.64–1.36)	0.715	0.876
Art, music, or dance	Expectancy	59/1,496	0.82 (0.50–1.35)	0.440	0.786
Traditional healing systems	Favorability	22/306	1.23 (0.55–2.76)	0.613	0.876
Traditional healing systems	Expectancy	145/2,716	0.76 (0.56–1.03)	0.080	0.786
Visit to a naturopathic physician	Favorability	21/156	1.45 (0.60–3.52)	0.409	0.818
Visit to a naturopathic physician	Expectancy	146/2,866	0.98 (0.72–1.32)	0.878	0.999
Heat or cold therapy	Favorability	90/1,073	0.83 (0.56–1.23)	0.353	0.514
Heat or cold therapy	Expectancy	77/1,949	1.04 (0.69–1.58)	0.837	0.917
Over-the-counter medication	Favorability	149/2,495	0.59 (0.44–0.81)	<0.001	0.006
Over-the-counter medication	Expectancy	18/527	0.73 (0.30–1.75)	0.473	0.917
Physical therapy	Favorability	61/646	0.90 (0.55–1.47)	0.683	0.797
Physical therapy	Expectancy	106/2,376	0.83 (0.59–1.18)	0.301	0.917
Psychotherapy	Favorability	35/336	1.83 (0.94–3.56)	0.075	0.251
Psychotherapy	Expectancy	132/2,686	1.02 (0.74–1.39)	0.917	0.917
Prescription diet	Favorability	48/397	1.59 (0.90–2.80)	0.108	0.251
Prescription diet	Expectancy	119/2,625	0.95 (0.68–1.33)	0.773	0.917
Prescription medication	Favorability	150/2,204	0.98 (0.73–1.32)	0.895	0.895
Prescription medication	Expectancy	17/818	0.33 (0.15–0.76)	0.009	0.063
Surgery	Favorability	42/446	0.77 (0.44–1.36)	0.367	0.514
Surgery	Expectancy	125/2,576	1.07 (0.78–1.47)	0.667	0.917

Table S5. Adjusted differences in undichotomized respondent-level category and overall composite means.

Linear models use the original covariates, HC3 standard errors, and t-based confidence intervals. Differences are reported CFS minus the comparison group on the 1–6 coded scale. q uses separate six-test favorability and expectancy families. Respondent means contain different sets of approaches and assume equal spacing of response levels.

Composite	Outcome	CFS n/total	Comparison mean	Reported CFS mean	Adjusted difference (95% CI)	p	q
Overall CHA	Favorability	164/2,864	4.913	4.771	-0.134 (-0.282 to 0.014)	0.075	0.225
Overall CHA	Expectancy	167/3,017	3.030	2.999	-0.030 (-0.220 to 0.160)	0.755	0.755
Nutritional	Favorability	150/2,438	4.696	4.644	-0.004 (-0.180 to 0.172)	0.963	0.963
Nutritional	Expectancy	160/2,981	3.211	3.059	-0.146 (-0.352 to 0.060)	0.165	0.728
Physical	Favorability	74/837	4.910	5.020	0.119 (-0.154 to 0.393)	0.393	0.471
Physical	Expectancy	166/3,009	3.204	3.274	0.058 (-0.156 to 0.272)	0.594	0.728
Psychological	Favorability	132/2,165	5.103	4.814	-0.289 (-0.512 to -0.066)	0.011	0.068
Psychological	Expectancy	165/2,995	2.667	2.738	0.071 (-0.145 to 0.287)	0.518	0.728
Combined	Favorability	138/2,168	5.000	4.899	-0.103 (-0.280 to 0.075)	0.257	0.385
Combined	Expectancy	165/3,012	3.063	2.970	-0.085 (-0.289 to 0.119)	0.413	0.728
Systems	Favorability	33/372	4.406	4.621	0.238 (-0.139 to 0.615)	0.215	0.385
Systems	Expectancy	157/2,932	2.962	2.889	-0.063 (-0.304 to 0.177)	0.606	0.728

Table S6. Composite-rating sensitivity to exact-midpoint handling under matched six-test families.

The same six-test rating-outcome families are used for both cutoffs, so this comparison isolates treatment of exact midpoint means. The revised analysis defines only means >3.5 as positive. A mean exactly 3.5 can represent mixed ratings. Midpoint n/CFS gives all exact ties and ties among respondents reporting CFS. Individual integer ratings are unchanged.

Composite	Outcome	≥3.5 aOR (95% CI)	q	>3.5 aOR (95% CI)	q	Midpoint n/CFS
Overall CHA	Favorability	0.71 (0.39–1.29)	0.515	0.67 (0.40–1.14)	0.216	41/4
Overall CHA	Expectancy	1.10 (0.79–1.52)	0.696	1.06 (0.76–1.47)	0.847	12/2
Nutritional	Favorability	1.28 (0.70–2.32)	0.568	1.16 (0.68–1.98)	0.579	48/4
Nutritional	Expectancy	0.82 (0.59–1.14)	0.483	0.76 (0.55–1.07)	0.694	128/9
Physical	Favorability	1.26 (0.54–2.92)	0.588	1.57 (0.68–3.61)	0.344	15/0
Physical	Expectancy	0.91 (0.66–1.26)	0.696	0.97 (0.70–1.34)	0.847	43/0
Psychological	Favorability	0.53 (0.30–0.94)	0.176	0.46 (0.28–0.75)	0.013	33/6
Psychological	Expectancy	1.05 (0.74–1.49)	0.781	1.10 (0.77–1.57)	0.847	64/2
Combined	Favorability	0.78 (0.39–1.54)	0.568	0.64 (0.36–1.16)	0.216	24/4
Combined	Expectancy	0.81 (0.58–1.13)	0.483	0.88 (0.63–1.23)	0.847	70/1
Systems	Favorability	2.51 (0.78–12.76)	0.403	2.93 (0.91–14.83)	0.216	7/0
Systems	Expectancy	0.80 (0.57–1.11)	0.483	0.93 (0.66–1.31)	0.847	240/7

Table S7. Exposure-focused proportional-odds diagnostics and reasons for nonassessment.

An exposure-focused partial-proportional-odds likelihood comparison was attempted only if every diagnosis-by-rating cell had at least five observations. The screen permitted 23 expectancy diagnostics and excluded 47 models, including every favorability model. Diagnostic p-values are nominal, without multiplicity correction, and do not assess proportional odds for every covariate. A dash denotes a diagnostic not assessed under the screen.

Approach	Outcome	Diagnostic status	LR statistic	df	Nominal p
Vitamin or mineral supplements	Favorability	Not assessed: sparse cells	—	—	—
Vitamin or mineral supplements	Expectancy	Not assessed: sparse cells	—	—	—
Other dietary supplements or natural products	Favorability	Not assessed: sparse cells	—	—	—
Other dietary supplements or natural products	Expectancy	Assessed	2.772	4	0.597
Herbalist consultation	Favorability	Not assessed: sparse cells	—	—	—
Herbalist consultation	Expectancy	Assessed	1.548	4	0.818
Self-directed special diet	Favorability	Not assessed: sparse cells	—	—	—
Self-directed special diet	Expectancy	Assessed	1.645	4	0.801
Visit to a homeopathic practitioner	Favorability	Not assessed: sparse cells	—	—	—
Visit to a homeopathic practitioner	Expectancy	Assessed	0.783	4	0.941
Chiropractic care	Favorability	Not assessed: sparse cells	—	—	—
Chiropractic care	Expectancy	Assessed	3.823	4	0.431
Osteopathic manipulation	Favorability	Not assessed: sparse cells	—	—	—
Osteopathic manipulation	Expectancy	Assessed	11.879	4	0.018
Massage therapy	Favorability	Not assessed: sparse cells	—	—	—
Massage therapy	Expectancy	Assessed	11.285	4	0.024
Light, magnetic, or electrical approaches other than physical therapy	Favorability	Not assessed: sparse cells	—	—	—
Light, magnetic, or electrical approaches other than physical therapy	Expectancy	Assessed	4.656	4	0.324
Other nonconventional devices	Favorability	Not assessed: sparse cells	—	—	—
Other nonconventional devices	Expectancy	Assessed	2.615	4	0.624
Spiritual healing	Favorability	Not assessed: sparse cells	—	—	—
Spiritual healing	Expectancy	Assessed	0.580	4	0.965
Prayer	Favorability	Not assessed: sparse cells	—	—	—
Prayer	Expectancy	Not assessed: sparse cells	—	—	—
Mindfulness or spiritual practices other than prayer	Favorability	Not assessed: sparse cells	—	—	—
Mindfulness or spiritual practices other than prayer	Expectancy	Not assessed: sparse cells	—	—	—
Shamanic journey	Favorability	Not assessed: sparse cells	—	—	—
Shamanic journey	Expectancy	Not assessed: sparse cells	—	—	—
Guided hypnotherapy	Favorability	Not assessed: sparse cells	—	—	—
Guided hypnotherapy	Expectancy	Assessed	6.791	4	0.147
Self-hypnosis	Favorability	Not assessed: sparse cells	—	—	—
Self-hypnosis	Expectancy	Not assessed: sparse cells	—	—	—
Deep breathing exercises	Favorability	Not assessed: sparse cells	—	—	—
Deep breathing exercises	Expectancy	Not assessed: sparse cells	—	—	—
Progressive relaxation	Favorability	Not assessed: sparse cells	—	—	—
Progressive relaxation	Expectancy	Assessed	7.034	4	0.134
Meditation	Favorability	Not assessed: sparse cells	—	—	—
Meditation	Expectancy	Not assessed: sparse cells	—	—	—
Biofeedback	Favorability	Not assessed: sparse cells	—	—	—
Biofeedback	Expectancy	Assessed	3.037	4	0.552

Approach	Outcome	Diagnostic status	LR statistic	df	Nominal p
Guided imagery	Favorability	Not assessed: sparse cells	—	—	—
Guided imagery	Expectancy	Not assessed: sparse cells	—	—	—
Acupuncture	Favorability	Not assessed: sparse cells	—	—	—
Acupuncture	Expectancy	Assessed	5.174	4	0.270
Yoga	Favorability	Not assessed: sparse cells	—	—	—
Yoga	Expectancy	Assessed	3.982	4	0.408
Tai chi or qigong	Favorability	Not assessed: sparse cells	—	—	—
Tai chi or qigong	Expectancy	Assessed	2.429	4	0.657
Movement therapy	Favorability	Not assessed: sparse cells	—	—	—
Movement therapy	Expectancy	Assessed	2.147	4	0.709
Art, music, or dance	Favorability	Not assessed: sparse cells	—	—	—
Art, music, or dance	Expectancy	Assessed	5.127	4	0.274
Traditional healing systems	Favorability	Not assessed: sparse cells	—	—	—
Traditional healing systems	Expectancy	Not assessed: sparse cells	—	—	—
Visit to a naturopathic physician	Favorability	Not assessed: sparse cells	—	—	—
Visit to a naturopathic physician	Expectancy	Assessed	0.502	4	0.973
Heat or cold therapy	Favorability	Not assessed: sparse cells	—	—	—
Heat or cold therapy	Expectancy	Assessed	3.088	4	0.543
Over-the-counter medication	Favorability	Not assessed: sparse cells	—	—	—
Over-the-counter medication	Expectancy	Not assessed: sparse cells	—	—	—
Physical therapy	Favorability	Not assessed: sparse cells	—	—	—
Physical therapy	Expectancy	Assessed	1.774	4	0.777
Psychotherapy	Favorability	Not assessed: sparse cells	—	—	—
Psychotherapy	Expectancy	Not assessed: sparse cells	—	—	—
Prescription diet	Favorability	Not assessed: sparse cells	—	—	—
Prescription diet	Expectancy	Assessed	2.834	4	0.586
Prescription medication	Favorability	Not assessed: sparse cells	—	—	—
Prescription medication	Expectancy	Not assessed: sparse cells	—	—	—
Surgery	Favorability	Not assessed: sparse cells	—	—	—
Surgery	Expectancy	Assessed	10.004	4	0.040

Table S8. Number and composition of ratings contributing to respondent-level composites.

CFS 1 denotes reported clinician diagnosis and CFS 0 no reported diagnosis. Rating counts describe the available approaches contributing to each respondent-level mean. ρ is the within-group, unadjusted Spearman correlation between rating count and mean score. The final column counts means exactly 3.5. These are descriptive quantities, without hypothesis-test claims.

Composite	Outcome	CFS	N	Mean count	Median count (IQR)	Count range	Mean score (SD)	ρ	At 3.5
Overall CHA	Favorability	0	2,700	5.69	5.0 (3.0–8.0)	1–28	4.91 (0.91)	-0.019	37
Overall CHA	Favorability	1	164	7.46	6.0 (4.0–10.0)	1–27	4.77 (0.95)	0.135	4
Overall CHA	Expectancy	0	2,850	22.66	24.0 (21.0–26.0)	1–28	3.03 (1.21)	-0.297	10
Overall CHA	Expectancy	1	167	20.68	22.0 (18.5–24.0)	1–28	3.00 (1.23)	-0.436	2
Nutritional	Favorability	0	2,288	1.82	2.0 (1.0–2.0)	1–5	4.70 (1.12)	-0.033	44
Nutritional	Favorability	1	150	2.06	2.0 (1.0–3.0)	1–5	4.64 (1.05)	0.173	4
Nutritional	Expectancy	0	2,821	3.59	4.0 (3.0–4.0)	1–5	3.21 (1.29)	-0.162	119
Nutritional	Expectancy	1	160	3.29	3.0 (3.0–4.0)	1–5	3.06 (1.30)	-0.261	9
Physical	Favorability	0	763	1.59	1.0 (1.0–2.0)	1–5	4.91 (1.22)	-0.127	15
Physical	Favorability	1	74	1.93	2.0 (1.0–3.0)	1–5	5.02 (1.12)	-0.285	0
Physical	Expectancy	0	2,843	4.59	5.0 (4.0–5.0)	1–5	3.20 (1.37)	-0.164	43
Physical	Expectancy	1	166	4.17	5.0 (3.2–5.0)	1–5	3.27 (1.36)	-0.193	0
Psychological	Favorability	0	2,033	1.70	1.0 (1.0–2.0)	1–6	5.10 (1.11)	-0.118	27
Psychological	Favorability	1	132	1.89	2.0 (1.0–2.0)	1–6	4.81 (1.27)	-0.159	6
Psychological	Expectancy	0	2,830	4.83	5.0 (4.0–6.0)	1–6	2.67 (1.34)	-0.196	62
Psychological	Expectancy	1	165	4.56	5.0 (4.0–5.0)	1–6	2.74 (1.38)	-0.378	2
Combined	Favorability	0	2,030	3.02	3.0 (1.0–4.0)	1–10	5.00 (0.99)	0.015	20
Combined	Favorability	1	138	3.47	3.0 (2.0–5.0)	1–10	4.90 (1.02)	0.106	4
Combined	Expectancy	0	2,847	7.88	8.0 (7.0–10.0)	1–10	3.06 (1.32)	-0.244	69
Combined	Expectancy	1	165	7.22	7.0 (6.0–9.0)	1–10	2.97 (1.31)	-0.325	1
Systems	Favorability	0	339	1.24	1.0 (1.0–1.0)	1–2	4.41 (1.29)	0.053	7
Systems	Favorability	1	33	1.30	1.0 (1.0–2.0)	1–2	4.62 (0.97)	0.167	0
Systems	Expectancy	0	2,775	1.91	2.0 (2.0–2.0)	1–2	2.96 (1.49)	-0.136	233
Systems	Expectancy	1	157	1.85	2.0 (2.0–2.0)	1–2	2.89 (1.49)	-0.316	7

Table S9. Descriptive summaries and use associations after separate additional adjustment for other reported diagnoses or physician-visit category.

Panel A describes the additional summary variables. Panel B compares the original adjustment set with two separate augmented specifications. All use models have N=3,022. q uses a six-test CHA-use family or a seven-test conventional-use family within each augmented specification. The count includes 18 potentially overlapping self-reported clinician diagnoses and is not a validated morbidity or severity index. These conditional associations do not establish causal effects, temporal order, or why estimates change.

Panel A. Additional-summary descriptives

Summary	CFS	N	Value
Other reported diagnoses	0	2,855	Mean 2.19; median 2 (1–3)
Physician visits: 0	0	2,855	539 (18.9%)
Physician visits: 1–2	0	2,855	1,002 (35.1%)
Physician visits: 3–5	0	2,855	836 (29.3%)
Physician visits: 6–9	0	2,855	278 (9.7%)
Physician visits: ≥10	0	2,855	200 (7.0%)
Other reported diagnoses	1	167	Mean 5.67; median 5 (3–7)
Physician visits: 0	1	167	8 (4.8%)
Physician visits: 1–2	1	167	44 (26.3%)
Physician visits: 3–5	1	167	53 (31.7%)
Physician visits: 6–9	1	167	30 (18.0%)
Physician visits: ≥10	1	167	32 (19.2%)

Panel B. Adjusted use associations

Use outcome	Original aOR (95% CI)	Plus diagnosis count	q	Plus visit category	q
Overall CHA	2.46 (0.97–8.91)	1.32 (0.50–4.89)	0.726	1.63 (0.63–5.96)	0.347
Nutritional	2.25 (1.34–3.80)	1.66 (0.97–2.85)	0.369	1.80 (1.06–3.05)	0.044
Physical	2.22 (1.60–3.07)	1.27 (0.89–1.82)	0.369	1.86 (1.33–2.59)	0.001
Psychological	1.44 (0.98–2.13)	1.00 (0.66–1.50)	0.984	1.32 (0.89–1.95)	0.206
Combined	1.89 (1.24–2.88)	1.35 (0.87–2.09)	0.369	1.71 (1.12–2.62)	0.027
Systems	1.99 (1.32–3.01)	1.24 (0.79–1.96)	0.526	1.74 (1.15–2.65)	0.027
Heat or cold therapy	2.18 (1.58–3.00)	1.10 (0.77–1.57)	0.806	1.87 (1.35–2.60)	<0.001
Over-the-counter medication	1.67 (1.00–2.78)	1.14 (0.67–1.93)	0.806	1.40 (0.84–2.35)	0.199
Physical therapy	2.22 (1.59–3.10)	1.05 (0.72–1.53)	0.806	1.60 (1.13–2.27)	0.013
Psychotherapy	2.22 (1.48–3.33)	1.38 (0.88–2.15)	0.520	1.73 (1.14–2.61)	0.013
Prescription diet	2.88 (2.00–4.15)	1.47 (0.97–2.22)	0.464	2.36 (1.63–3.42)	<0.001
Prescription medication	3.46 (2.05–5.84)	1.42 (0.81–2.50)	0.520	2.27 (1.29–4.02)	0.011
Surgery	2.04 (1.41–2.96)	1.06 (0.69–1.62)	0.806	1.48 (1.00–2.17)	0.057

Figure S1. Overall and category CHA-use estimates under the original covariates, with added other-condition count, and with added physician-visit category. CFS refers to a self-reported clinician diagnosis. Horizontal bars are 95% CIs; OR >1 indicates higher odds of reported use. The post hoc specifications do not identify causal effects or establish control of symptom severity or diagnosis ascertainment.

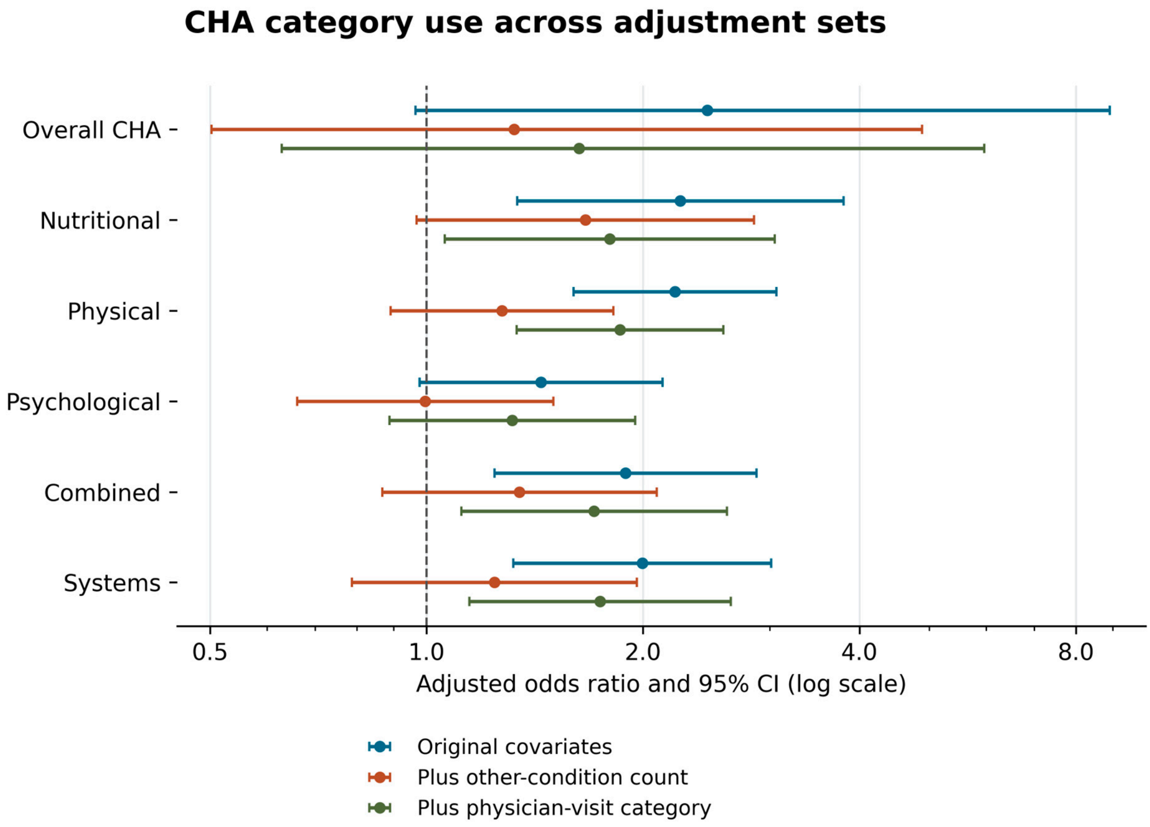

Supplement: Supplementary file 1 [file jcm-15-08425-s001.zip › jcm-4605244-supplementary.pdf]
